# *Phytophthora* root rot induces compositional and functional changes in avocado rhizosphere bacterial communities

**DOI:** 10.1093/femsmc/xtag025

**Published:** 2026-05-12

**Authors:** Rosaura G Alfaro-García, Pablo Vargas-Mejía, Violeta Patiño-Conde, Eria A Rebollar, José A Guerrero-Analco, Julio Vega-Arreguín, Frédérique Reverchon, Alfonso Méndez-Bravo

**Affiliations:** Red de Diversidad Biológica del Occidente Mexicano, Instituto de Ecología, A. C., Centro Regional del Bajío, Av. Lázaro Cárdenas 253, Centro, 61600 Pátzcuaro, México; Laboratorio Nacional de Análisis y Síntesis Ecológica, Escuela Nacional de Estudios Superiores, Unidad Morelia, Universidad Nacional Autónoma de México, Antigua carretera a Pátzcuaro 8701, Ex Hacienda de San José de la Huerta, 58190 Morelia, México; Laboratorio de Ciencias Agrogenómicas and Laboratorio Nacional PlanTECC, Universidad Nacional Autónoma de México, Blvd. UNAM 2011, 37684 León, México; Laboratorio Nacional de Análisis y Síntesis Ecológica, Escuela Nacional de Estudios Superiores, Unidad Morelia, Universidad Nacional Autónoma de México, Antigua carretera a Pátzcuaro 8701, Ex Hacienda de San José de la Huerta, 58190 Morelia, México; Centro de Ciencias Genómicas, Universidad Nacional Autónoma de México, Av. Universidad s/n, Universidad Autónoma del Estado de Morelos, 62210 Cuernavaca, México; Red de Estudios Moleculares Avanzados, Instituto de Ecología, A. C., Carretera Antigua a Coatepec 351, El Haya, 91073 Xalapa, México; Laboratorio de Ciencias Agrogenómicas and Laboratorio Nacional PlanTECC, Universidad Nacional Autónoma de México, Blvd. UNAM 2011, 37684 León, México; Red de Diversidad Biológica del Occidente Mexicano, Instituto de Ecología, A. C., Centro Regional del Bajío, Av. Lázaro Cárdenas 253, Centro, 61600 Pátzcuaro, México; Laboratorio Nacional de Análisis y Síntesis Ecológica, Escuela Nacional de Estudios Superiores, Unidad Morelia, Universidad Nacional Autónoma de México, Antigua carretera a Pátzcuaro 8701, Ex Hacienda de San José de la Huerta, 58190 Morelia, México; SECIHTI, Ciudad de México, México

**Keywords:** Dysbiosis, Metatranscriptomics, *Persea americana*, *Phytophthora cinnamomi*, Rhizobacteria, Stress response

## Abstract

Understanding how plant pathogens modulate the rhizosphere microbiota is essential to integrated disease management. Here, the compositional and functional shifts in the avocado rhizosphere bacteriome induced by *Phytophthora cinnamomi* were assessed to identify bacterial taxa enriched in the symptomatic condition and elucidate the microbial functions modulated by the infection. Metabarcoding and metatranscriptomics analyses revealed that *Phytophthora* root rot (PRR) induced compositional shifts in bacterial communities, leading to the enrichment of members of MND1, RB41, and *Nitrospira*. Functional analysis showed that this enrichment might be due to the release of nutrients following root rot, as carbohydrate metabolism was stimulated in rhizobacterial communities of infected trees. Moreover, the relative abundance of transcripts from genes associated with stress response and cell signaling increased in some of the most active genera in the rhizosphere of PRR-symptomatic trees, suggesting their potential to mitigate the adverse effects of infection. These findings highlight the need to combine compositional and functional microbiome data to differentiate between taxa attracted by nutrient release and those contributing to the plant defense. The interactions of beneficial bacterial taxa with the pathogen should be further studied, as they may constitute promising biocontrol agents.

## Teaser

Compositional and functional readjustments of the rhizobacterial community in avocado induced by Phytophthora root rot.

## Introduction

The rhizosphere microbiota is critical for plant health and productivity (Trivedi et al. 2020). By improving plant nutrient uptake, producing phytohormone-like compounds, stimulating their host plant defense response, and increasing their tolerance to abiotic and biotic stressors, rhizosphere microbial communities contribute to their host´s growth and fitness (Méndez-Bravo et al. 2018, Compant et al. 2025). Research from the last two decades has shed light on the factors driving the assembly of rhizosphere microbial communities, which are both host-dependent (e.g. plant genotype or developmental stage; Chaparro et al. 2013, Philippot et al. 2013, Mendes et al. 2018) and host-independent (e.g. soil parameters, climatic variables, or human management practices; Hartmann et al. 2014, Carrasco-Espinosa et al. 2022, Santoyo 2022). In particular, plant health status has been shown to be a strong determinant of rhizosphere microbial assemblages, as plant infection by pathogens can induce shifts in diversity, composition, and function in plant-associated microbiota, leading to a possible dysbiotic state (Berendsen et al. 2012, Alfaro-García et al. 2026). In light of the plant holobiont theory (Vandenkoornhuyse et al. 2015), understanding how plant pathogens modulate the rhizosphere microbiota may hold the key to more efficient strategies for integral disease management, which is particularly relevant in monoculture cropping systems where soil-borne pathogen loads may be accrued (Hiddink et al. 2010, Dou et al. 2025).

Soil-borne pathogens produce contrasting effects on rhizosphere microbial diversity and structure. Depending on the pathosystem under study, pathogens may increase (Mendes et al. 2023) or decrease (Wei et al. 2018, Zhou et al. 2021) the richness and diversity of rhizosphere microbial communities, and sometimes appear not to affect microbial diversity (Hassani et al. 2023, Guerrero et al. 2025). The effects of soil-borne pathogens on the structure of rhizosphere microbial communities are more consistent, as microbial community structure in the rhizosphere is usually altered by soil-borne pathogens (Alfaro-García et al. 2026). Competition of the rhizosphere microbiota with pathogen populations and/or the secretion of effectors by the pathogens reorganize microbial interactions in the rhizosphere (Wei et al. 2018, Snelders et al. 2022). Moreover, the activation of the plant immune response upon infection leads to shifts in root exudates and plant—microbe interactions, enriching the rhizosphere in those microbial taxa able to metabolize such compounds (Gu et al. 2016, Yang et al. 2023). At the functional level, soil-borne pathogens modulate some of the functions performed by the rhizosphere microbiota, e.g. by activating the expression of genes related with stress and defense response (Radl et al. 2019, Batista et al. 2024) or by reducing the expression of genes linked with autotoxin degradation (Wen et al. 2022). Although such functional insights are still scarcely investigated, they contribute to a better understanding of the dysbiosis caused by soil-borne pathogens and ultimately may assist in the identification of markers of disease suppression (Alfaro-García et al. 2026).

*Phytophthora cinnamomi* Rands., a soil-borne oomycete, is considered one of the most devastating pathogens worldwide (Hardham and Blackman 2018)). Root rot caused by *P. cinnamomi* causes severe ecological and economic damage in a wide range of natural and agro-ecosystems (Shands et al. 2025). In avocado (*Persea americana* Mill.), *Phytophthora* root rot (PRR) represents the major limiting factor of the fruit global production, including Mexico, the world´s leading producer and exporter of avocado (Fernández-Pavía et al. 2013, Guevara-Avendaño et al. 2024). The shifts in the avocado rhizosphere microbiome induced by *P. cinnamomi* have been studied at the compositional and structural level, with contrasting results. Yang et al. (2001), using denaturing gradient gel electrophoresis (DGGE), detected that the rhizosphere bacterial community of avocado was more diverse in roots infected with *P. cinnamomi* than in uninfected roots, although uninfected roots were colonized by bacterial genera such as *Pseudomonas, Polyangium*, and *Cytophaga*, which have been reported as controllers of pathogens (Yuan et al. 2022, Lv et al. 2023). Using shotgun metagenomics, Shu et al. (2019) reported that the avocado rhizosphere microbiota was modified by PRR, and showed an increase in the relative abundance of the ten most representative bacterial taxa in infected plants compared with uninfected trees. More recently, Solís-García et al. (2021) used a metabarcoding approach to report shifts in the composition of avocado bacterial and fungal communities due to PRR, with an increase in the relative abundance of copiotrophic taxa such as Pseudomonadales and Burkholderiales and a concomitant decrease of Actinobacteria and Rhizobiales in the rhizosphere of infected trees. Considering the heterogeneity of the described shifts in avocado rhizobacterial communities driven by PRR, further research should focus on the functional consequences of the disease, to elucidate the microbial processes enhanced or hindered by the infection and identify potential contributions of the rhizosphere microbiome in the plant defense response (Flores-Nunez and Stukenbrock 2024, Alfaro-García et al. 2026).

In this study, the impact of PRR on the diversity, composition, and gene expression profile of avocado rhizosphere bacterial communities was studied in an orchard located in the world´s leading producing region. By combining a metabarcoding approach with a metatranscriptomic analysis of the rhizosphere bacteriome in asymptomatic and PRR-symptomatic avocado trees, we sought to identify potential bacterial taxa and functional markers associated with the disease or contributing to its mitigation. Based on findings from previous studies investigating the effects of root rot on rhizosphere microbiomes (Shu et al. 2019, Solís-García et al. 2021, Magagula et al. 2025), we hypothesized that (1) symptomatic trees would exhibit an increased dominance of copiotrophic bacterial taxa in their rhizosphere due to root necrosis, which would be reflected at the compositional and functional level, and (2) PRR would induce the expression of stress-related genes in rhizosphere bacterial communities of symptomatic trees.

## Materials and methods

### Study site and rhizosphere soil sampling

The study orchard was located in Peribán, Michoacán (19° 31′16′′ N, 102° 24′54′′ W) at an altitude of 1640 m.a.s.l. The mean annual temperature is 19.8°C, with an annual precipitation of 1445 mm, mostly falling between June and September (CONAGUA, 2023). The selected orchard covered an area of six hectares and had an organic management. Avocado trees (Méndez cultivar) were aged between eight and 15 years old. The presence of *P. cinnamomi* has been recorded in recent years in the orchard. In April 2023, 10 avocado trees showing symptoms of PRR according to Solís-García et al. (2021)—with defoliation, dead or chlorotic leaves, coupled with small, brittle and/or necrotic roots—were randomly selected, as well as 10 asymptomatic avocado trees, spanning the entire orchard. All trees were at least 20 m apart. The presence of *P. cinnamomi* in the roots of PRR-symptomatic trees was confirmed by culture-dependent methods. Around each selected tree, a circumference no greater than 100 cm was drawn and the rhizosphere soil adhered to fine roots was collected at four points around the trunk by digging with an ethanol-disinfected shovel (10 cm depth). The four samples were mixed and homogenized to obtain one rhizosphere soil sample per tree, and subsequently divided into two centrifuge tubes (50 ml), one for DNA extraction and the other for RNA extraction. 30 ml of Qiagen’s LifeGuard® Soil Preservation Solution was added to the soil samples aimed for RNA extraction. All rhizosphere soil samples were kept cold with frozen gel bags until the arrival at the lab, where they were stored at -80°C until DNA and RNA extraction.

### Soil chemical characterization

Three randomly selected trees per condition (asymptomatic and PRR-symptomatic) were chosen in different areas of the orchard to collect 500 g of rhizosphere soil (*sensu lato, i.e*. soil around the fine roots of the tree) at approximately 100 cm away from the tree trunk, for background chemical characterization. Soil pH was measured in a 1:2 ratio in deionized water (AS-02-NOM-021-RECNAT-2000, SEMARNAT 2002). Electrical conductivity was registered in a 1:5 soil/water ratio (FAO 2021b). Available phosphorus (P) was determined with the Olsen method (FAO 2021a). Total carbon (C) and nitrogen (N) were quantified using the combustion method in an elemental analyzer (LECO, TRuspec model). The potassium chloride extraction method was used to determine nitrate (NO_3_^−^) and ammonium (NH_4_^+^) concentrations (Bremner 1965). All soil analyses were performed at the Laboratory for Soil Analyses of the *Instituto de Ecología, A.C*., Xalapa, México. A Student´s t-test was used to compare soil chemical characteristics between asymptomatic and PRR-symptomatic avocado trees. There were no significant differences in soil characteristics between samples from the rhizosphere of asymptomatic and PRR-symptomatic avocado trees (Table 1), except for NH_4_^+^, which was higher in the rhizosphere of asymptomatic trees than in that of PRR-symptomatic trees.

**Table 1:** Chemical characteristics (mean ± standard deviation, n = 3) of rhizosphere soil from asymptomatic and PRR-symptomatic avocado trees.

Condition	pH	EC	Available P	NH_4_^+^	NO_3_^−^	Total N	Total C	C/N
	1:2 H_2_O	mS/cm	mg/kg			%		ratio
Asymptomatic	6.49 ± 0.29	0.53 ± 0.37	178.25 ± 89.30	18.04 ± 2.07	30.90 ± 10.72	0.27 ± 0.11	3.60 ± 0.98	13.74 ± 1.74
Symptomatic	6.82 ± 0.14	0.26 ± 0.13	178.42 ± 85.72	12.97 ± 1.02	24.92 ± 7.67	0.30 ± 0.06	3.97 ± 0.96	13.38 ± 1.80
Student´s t-test	t = -1.74, df = 2.83, *p* = 0.18	t = -1.22, df = 2.46, *p* = 0.32	t = -0.002, df = 3.99, *p* = 0.99	t = 3.80, df = 2.92, *p* = 0.03*	t = 0.77, df = 3.62, *p* = 0.47	t = -0.38, df = 3.10, *p* = 0.72	t = -0.46, df = 3.99, *p* = 0.66	t = 0.25, df = 3.99, *p* = 0.81

### DNA extraction and construction of the 16S rRNA gene sequencing libraries

DNA extractions from rhizosphere soil were performed with the DNeasy® PowerSoil® Kit (Qiagen) following the manufacturer´s instructions. Purity and concentration of extracted DNA were verified using a BioSpectrometer® (Eppendorf). 20 libraries were constructed for taxonomic profiling of the bacterial communities following the workflow plan outlined in 16S Metagenomic Sequencing Library Preparation of Illumina (2013) with slight modifications. The primers 341F (5´-CCTACGGGNGGCWGCAG-3´) and 805R (5´-GACTACHVGGGTATCTAATCC-3´) were used to amplify the V3-V4 region of the 16S rRNA gene to create ∼460 bp amplicons (Illumina 2013). These first amplicons were dual-labeled using the Nextera XT Index kit^TM^ (Illumina). Both PCR reactions (50 µl) were performed following the manufacturer’s instructions for the Multiplex PCR (Qiagen), using a total of 30–40 ng of DNA per amplicon reaction and a total of 15 ng per index reaction. The PCR cycling parameters for amplicons and index PCR followed the Illumina (2013) protocol but included one enzyme activation step of 15 minutes at 95°C. Each PCR product was purified with ProNex® Size Selective Purification System following the manufacturer´s instructions. The libraries were sequenced paired-end (PE) with a read length of 2 × 300 bp using the Illumina MiSeq^TM^ system at CD Genomics, obtaining an average of 103 646 PE reads per sample.

### RNA extraction

Duplicate RNA extractions were carried out from all rhizosphere soil samples using the RNeasy® PowerSoil® Total RNA Kit by Qiagen. Prior to the first step of the extraction protocol, each sample and its duplicate were centrifuged at 5000 *g* for 5 min to remove the LifeGuard® Soil Preservation Solution as recommended by the manufacturer. The integrity of the extracted RNA was evaluated by the presence of ribosomal RNA (rRNA) bands by electrophoresis. RNA concentration and purity were measured on an Eppendorf BioSpectrometer®. The nine samples with intact RNA retrieved from asymptomatic trees were randomly distributed into three equimolar pools and the 10 samples retrieved from PRR-symptomatic trees were similarly distributed into three equimolar pools. Six libraries were then built from the six pools at Macrogen, Inc. All the pools received a DNase pre-treatment and, for construction of metatranscriptomic libraries, a NEBNext® rRNA Depletion Kit (Bacteria) was used for rRNA depletion. All libraries were sequenced PE with a read length of 2 × 100 bp using the Illumina NovaSeq 6000^TM^ system at Macrogen, Inc., obtaining an average of 99 056 583 PE reads per sample.

### Bioinformatic and statistical analysis for 16S rRNA sequences

#### Quality control and 16S rRNA gene amplicon analysis

DNA (16S rRNA) sequences were subjected to a pre-processing analysis to verify sequence quality using the FastQC software v.0.12.1 (Andrews 2010). The Trimmomatic software v.0.39 was used for removing universal Illumina adapters and low-quality reads (Bolger et al. 2014). The following parameters were used as filters: sequence quality > 30, minimum sequence length > 180 bp and max sequence length 240 bp. Reads were trimmed by 10 bases at the 5´-end and 3´-end. PANDAseq was used to merge PE reads of 16S rRNA gene (Masella et al. 2012).

The composition and structure of the rhizobacterial community were analyzed using the DADA2 pipeline in R v.4.3.3 (Callahan et al. 2016, R Core Team 2023). After merging, amplicons were trimmed again to 450 bp as minimum sequence length (filterAndTrim function, truncLen argument) and sequences with undetermined bases were removed (filterAndTrim function, maxN argument). Afterwards, sequences were de-replicated to reduce redundancy. An error model was generated by learning the specific error-signature for each sequence. After quality processing, sequences were grouped into Amplicon Sequence Variants (ASVs). Chimeras were removed before taxonomic assignment (removeBimeraDenovo function). Taxonomic assignment was performed using the SILVA v.138.1 database. Taxa with low prevalence (threshold ≤ 5%) were excluded from further analysis. Singletons, doubletons, mitochondria, chloroplast, and eukaryotic sequences were removed before diversity and statistical analyses.

#### Diversity and statistical analyses

The ASV table was rarefied to 36 227 reads using the rarefy_even_depth function implemented in the *Phyloseq* package v.1.46.0 (McMurdie and Holmes 2013) in R software. To calculate the alpha diversity indexes, the estimate_richness function of *Phyloseq* was used. Differences in Observed ASVs, Shannon and Simpson metrics between conditions were tested with the Mann-Whitney-Wilcoxon test using the wilcox.test function, indicating False in the paired argument. Venn diagrams were built to visualize the unique and shared bacterial ASVs in each condition with the *ggven* package in R (Gao et al. 2021), using all ASVs regardless of their abundance and, subsequently, considering only those taxa with a relative abundance larger than 0.005%, as recommended by Bokulich et al. (2013). For beta diversity analyses, the Bray-Curtis dissimilarity matrix was calculated from the normalized ASV table and used to build a non-metric multidimensional scaling (NMDS) using the ordinate and plot_ordination functions of *Phyloseq* package, respectively. A permutational multivariate analysis of variance (PERMANOVA) was used to compare the structure of the bacterial rhizosphere community of asymptomatic and PRR-symptomatic avocado trees, using the *VEGAN* package (Dixon 2003) with 999 permutations. To distinguish true biological differences from potential dispersion effects, we conducted a permutational analysis of multivariate dispersions (BETADISP) using the betadisper and permutest functions with 999 permutations in the R package *VEGAN*. The *DESeq2* package v.1.42.1 (Love et al. 2014) was used to compare the differential abundance of bacterial taxa between PRR-symptomatic and asymptomatic trees; significant differences were supported by a Wald test. All statistical analyses were performed using customized scripts with the free software R v.4.3.3 and were considered significant at *P* < 0.05. The analyses were visualized using the *ggplot2* v.4.0.0 package (Wickham 2016) in R software. The 16S rRNA gene amplicon libraries are available at the Sequence Read Archive of the NCBI under BioProject PRJNA1363127.

### Bioinformatic analysis of metatranscriptomic sequences

#### Quality control and preprocessing

Raw sequence libraries were checked for quality using the FastQC software. Adaptor trimming and removal of low-quality bases were performed with BBDuk from the BBMap suite v.39.06 using the parameters forcetrimleft = 13 and trimq = 10 to ensure high-confidence base calls (https://sourceforge.net/projects/bbmap/). Paired-end reads were subsequently merged using PEAR v.0.9.8 with default settings (Zhang et al. 2013). To eliminate rRNA, SortMeRNA v.4.3.6 (Kopylova et al. 2012) was employed using the SILVA rRNA databases for bacterial and archaeal 16S/23S, and eukaryotic 18S/28S subunits. The –num_alignments 1 parameter was used to retain only the best alignment per read. Reads identified as rRNA were removed for downstream analyses.

#### Read mapping, functional annotation, and quantification

Filtered reads were mapped to the NCBI RefSeq bacterial protein database (Tatusova et al. 2014) using DIAMOND v.2.1.6 (Buchfink et al. 2015) in blastx mode. Only the best-scoring alignment per read was retained to avoid redundant annotations. Alignment results were processed with the SAMSA2 pipeline (Westreich et al. 2018), which extracted protein-level matches and generated count matrices summarizing gene-, taxon- and function-level abundance for all metatranscriptomic libraries. To determine the taxonomic diversity of active bacterial communities, Shannon and Simpson indices were calculated with the diversity function of the *VEGAN* package in the R software. Statistical differences between conditions were tested utilizing the Wilcoxon test.

### Differential expression and functional enrichment analysis

The NCBI RefSeq bacterial protein database, used for read mapping with DIAMOND, was functionally annotated prior to analysis using EggNOG-mapper v.2 (Cantalapiedra et al. 2021) with the eggNOG v.5.0 database. This provided orthology assignments, functional descriptions, KEGG Orthology (KO) identifiers, and Gene Ontology (GO) terms for all reference proteins, enabling the functional interpretation of mapped reads. Read counts per gene were compiled from the SAMSA2 output and imported into *DESeq2* (Love et al. 2014) for differential expression analysis between asymptomatic and PRR-symptomatic avocado trees. Statistical significance was determined using the Wald test and adjusted for multiple testing using the Benjamini-Hochberg method. Functional enrichment analysis was performed using the *ClusterProfiler* R package (Yu et al. 2012), based on the GO and KEGG annotations assigned to the matched proteins in the annotated reference database. Metatranscriptomic libraries are available at the Sequence Read Archive of the NCBI under BioProject PRJNA1363127.

## Results

### Composition of the rhizosphere bacterial community in avocado trees

A total of 1 969 273 paired reads were obtained from 20 rhizosphere soil samples. After quality filtering, a total of 906 590 reads were retained, representing 25 862 bacterial ASVs. Rarefaction curves showed that rhizosphere samples from both asymptomatic and PRR-symptomatic avocado trees reached a plateau, indicating that most bacterial ASVs were detected (Supplementary Fig. 1).

**Figure 1:**
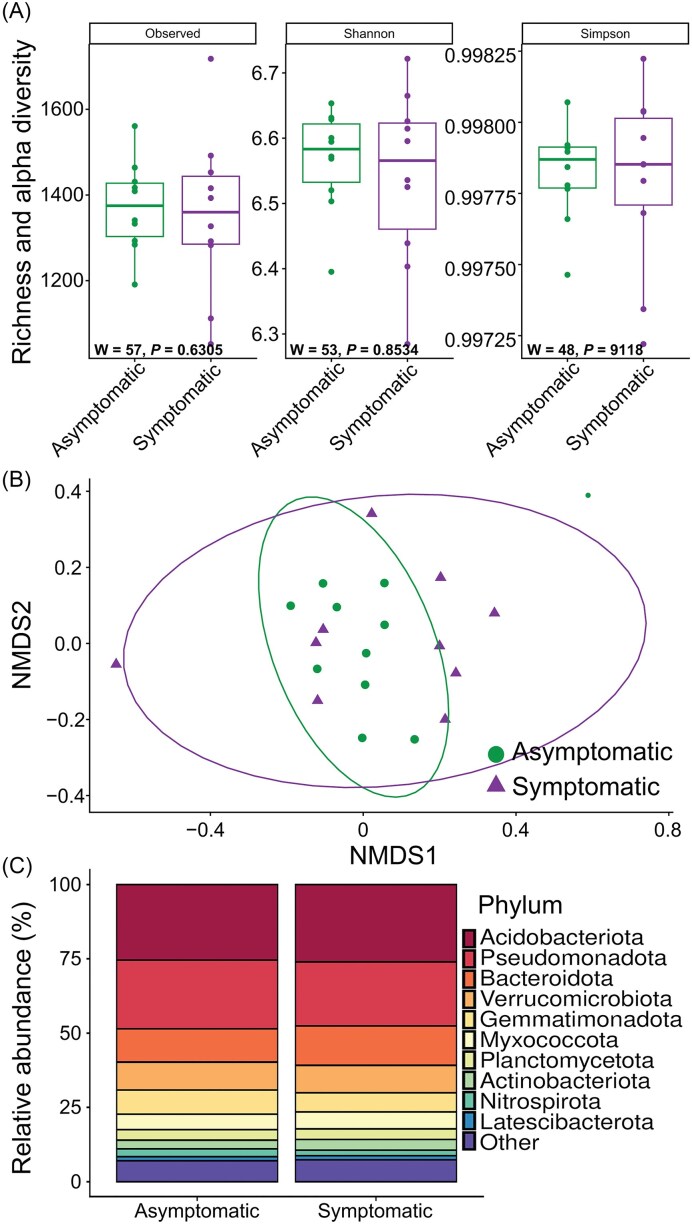
Diversity and composition of the bacterial community in the rhizosphere of asymptomatic and PRR-symptomatic avocado trees. (A) Observed richness, Shannon and Simpson indices of rhizobacterial communities associated with asymptomatic and PRR-symptomatic avocado trees. W and *P* values were calculated with the Mann-Whitney-Wilcoxon test (n = 10). (B) NMDS plot based on Bray-Curtis distance of the bacterial community associated with asymptomatic and PRR-symptomatic avocado trees (stress value = 0.19). (C) Relative abundance (%) of bacterial phyla associated with asymptomatic and PRR-symptomatic avocado trees. The “others” category represents phyla with a relative abundance < 1%.

The PRR infection did not induce significant differences in alpha diversity metrics (Observed richness, Shannon, and Simpson) of the rhizosphere bacterial communities compared to asymptomatic trees (Fig. 1A). Similarly, PRR did not affect the bacterial community structure in the avocado rhizosphere, as no significant differences were detected between samples from asymptomatic and PRR-symptomatic avocado trees (PERMANOVA df = 1, F = 2.88, *P* = 0.103; Fig. 1B). Interestingly, bacterial communities from PRR-symptomatic trees showed more dispersion than those associated with asymptomatic trees. Permutational analysis of multivariate dispersions (BETADISP, *P* = 0.09) confirmed that the observed dispersion could be driven by biological variation (*i.e*. presence or absence of the pathogen) rather than by differences in within-group dispersion.

At the compositional level, the five most abundant bacterial phyla were common to asymptomatic and PRR-symptomatic trees and were Acidobacteriota, Pseudomonadota, Bacteroidota, Verrucomicrobiota, and Gemmatimonadota, which together accounted for more than 70% of the total bacterial community. No significant differences were found in the relative abundance of the main bacterial phyla between conditions (Fig. 1C). However, at the genus level, compositional differences were observed between rhizosphere bacterial communities associated with asymptomatic and PRR-symptomatic trees.

**Figure 2:**
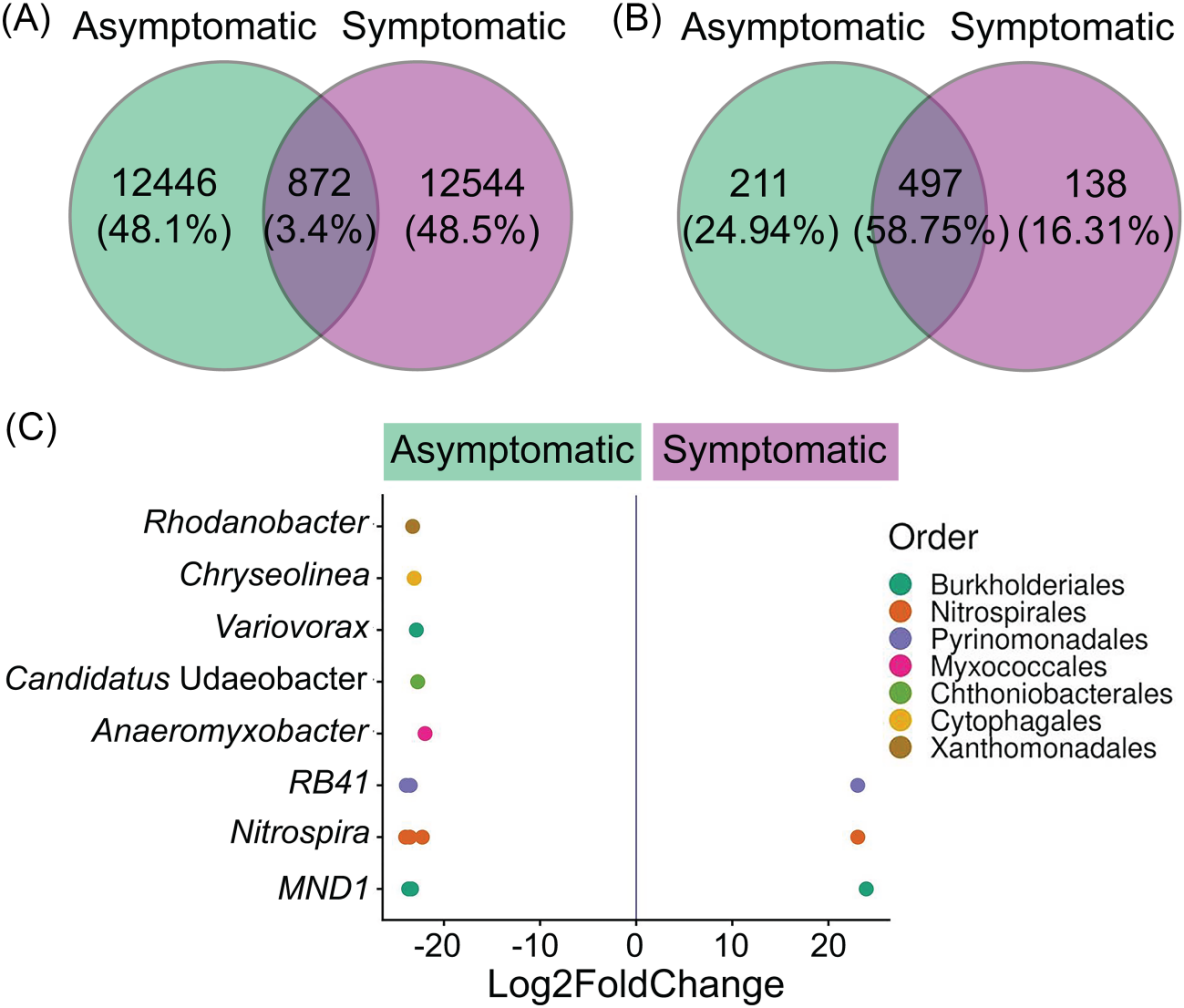
Shared, exclusive, and differentially abundant taxa in the rhizobacterial communities of asymptomatic and PRR-symptomatic avocado trees. (A) Venn diagram showing the number of unique and shared bacterial ASVs between the two tree conditions, considering all ASVs. (B) Venn diagram showing the number of unique and shared bacterial ASVs between the two tree conditions, considering only those ASVs with a relative abundance larger than 0.005%. (C) Differential abundance of bacterial ASVs identified at the genus level, associated with asymptomatic and PRR-symptomatic avocado trees, according to the Wald test (n = 10). Values of Log2FoldChange ≠ 0 and *P* < 0.05 are considered as differentially abundant in each condition.

From the 25 862 ASVs, only 872 (3.4%) were shared between the asymptomatic and PRR-symptomatic trees (Fig. 2A). When removing rare taxa (*i.e*. those whose relative abundance was ≤ 0.005%), the proportion of shared species increased to 58.75% (Fig. 2B). Differential abundances between conditions showed that there were more bacterial ASVs significantly enriched in asymptomatic than in PRR-symptomatic avocado trees (Fig. 2C). The *DeSeq* analysis supported by Wald test (*P* < 0.05) showed that rhizosphere bacterial ASVs affiliated with *Rhodanobacter, Chryoseolinea, Candidatus* Udaeobacter, *Anaeromyxobacter, Variovorax*, MND1, RB41, and *Nitrospira* were enriched in asymptomatic avocado trees. In contrast, other ASVs from MND1, RB41, and *Nitrospira* were significantly more abundant in the rhizosphere of PRR-symptomatic avocado trees.

### Functional diversity of the active rhizobacterial community of asymptomatic and PRR-symptomatic avocado trees

The composition of the active rhizosphere bacterial community was assessed using a comparative metatranscriptomic approach. Consistent with the results obtained from the metabarcoding data, alpha diversity indexes of the active bacterial communities did not vary between conditions (Supplementary Fig. 2A). Similarly, metatranscriptomic data obtained from asymptomatic and PRR-symptomatic rhizosphere soil samples did not display a clear separation in the Principal Component Analysis (PCA) (Supplementary Fig. 2B).

**Figure 3:**
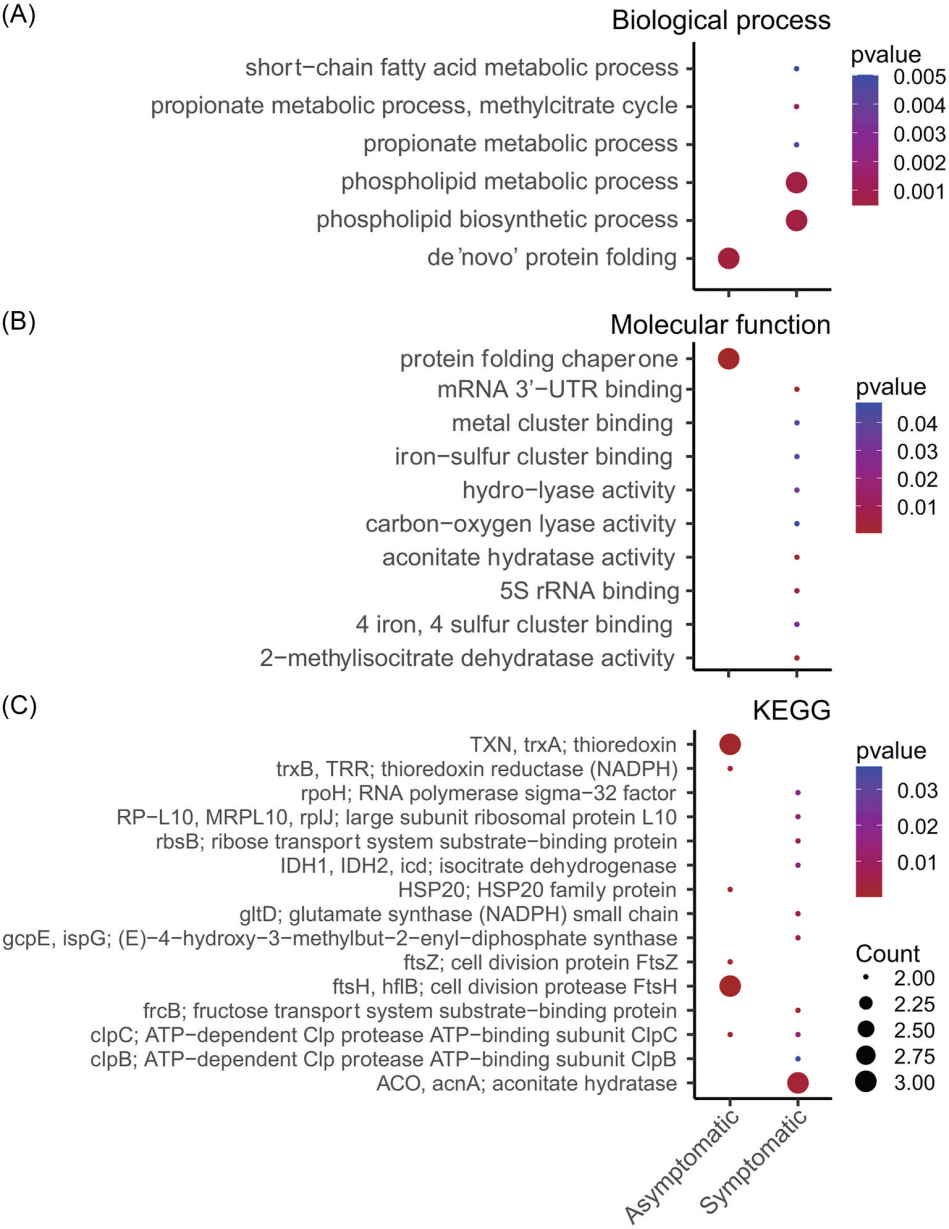
GO and KEGG analysis of the active bacterial community in the rhizosphere of asymptomatic and PRR-symptomatic avocado trees. Functional enrichment analysis of asymptomatic and PRR-symptomatic metatranscriptomes based on GO Biological process enrichment (A) and Molecular function enrichment (B) (n = 3 mRNA pools for each tree condition). (C) KO enrichment of asymptomatic and PRR-symptomatic metatranscriptomes. Dot size represents the number of differentially expressed genes within the enriched GO and KEGG categories and dot color represents the *P*-value (*P* < 0.05).

The differential expression analysis performed at the community level showed an increase in the relative abundance of 5496 transcripts in the rhizosphere of asymptomatic trees, whilst an increase in the relative abundance of 5891 transcripts was detected in the PRR-symptomatic trees. A gene enrichment analysis was performed to cluster transcripts into GO terms that describe Biological Processes (BP) and Molecular Functions (MF). Interestingly, the bacterial community in the rhizosphere of PRR-symptomatic avocado trees showed an enrichment in transcripts of BP (37 *vs*. 1) and MF (23 *vs*. 1) of GO compared to asymptomatic trees. For asymptomatic avocado trees, enriched transcripts were affiliated to “de novo” protein folding (GO0006458) and protein folding chaperone (GO0044183) in the BP and MF categories of GO, respectively (Fig. 3A and B). The most significantly enriched transcripts within BP in the PRR-symptomatic avocado trees were principally related to primary metabolism, such as phospholipid metabolic (GO0006644) and biosynthetic process (GO0008654), propionate metabolic process, methylcitrate process (GO0019679), and short-chain fatty acid metabolic process (GO0046459) (Fig. 3A). In the MF category, the transcripts enriched in the PRR-symptomatic trees were related to the mRNA 3´-UTR binding (GO0003730), aconitase hydratase activity (GO0003994), 5S rRNA binding (GO0008097), and 2-methylisocitrate dehydratase activity (GO0047456) (Fig. 3B).

Similarly, KO analysis revealed more functions performed by the bacterial community in the rhizosphere of PRR-symptomatic trees than those from asymptomatic trees (33 *vs*. 15). The most enriched functions in asymptomatic avocado trees were the thioredoxin trxA (K03671: Protein families-genetic information processing), cell division protease FtsH (K03798: Protein families-metabolism), and HSP20 family protein (K13993: Genetic information processing) (Supplementary Table S1). On the other hand, enriched functions in the PRR-symptomatic trees were related to aconitase hydratase (K01681: carbohydrate metabolism-citrate cycle (TCA)), RNA polymerase sigma-32 factor (K03089: genetic information processing-transcription machinery), large subunit ribosomal protein L10 (K02864: genetic information processing-translation), ribose transport system substrate-binding protein (K10439: environmental information processing-membrane transport), isocitrate dehydrogenase (K00031: carbohydrate metabolism-TCA), and glutamate synthase (NADPH) small chain (K00266: energy metabolism-nitrogen metabolism) (Fig. 3C; Supplementary Table S1).

### Composition and functional profile of active rhizobacteria

**Figure 4:**
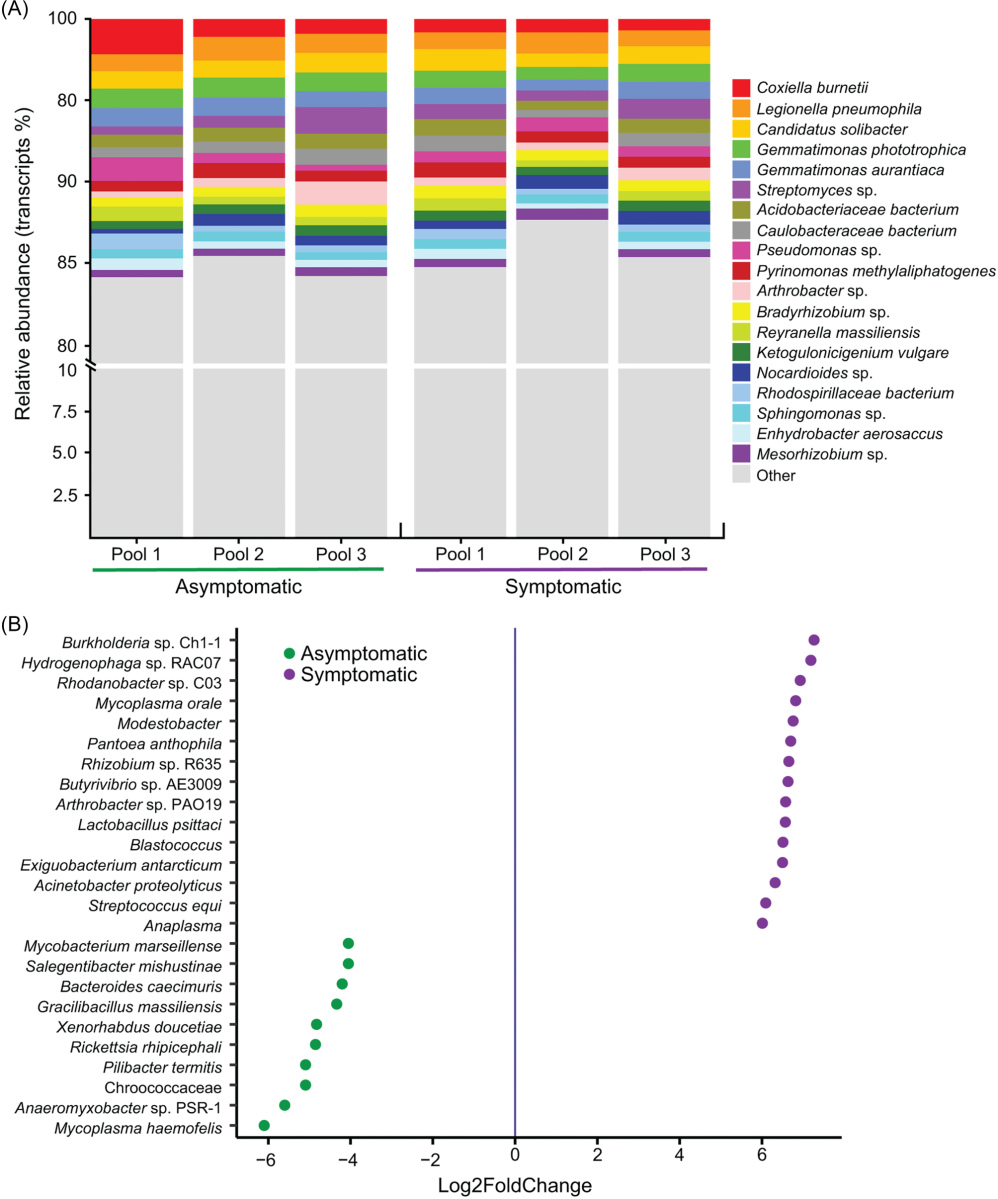
Taxonomic diversity of the active rhizobacterial community in the asymptomatic and PRR-symptomatic avocado trees. (A) Relative abundance (%) of the most abundant bacterial species associated with asymptomatic and PRR-symptomatic avocado trees as retrieved by metatranscriptome taxonomy assignments. The “others” category represents bacterial species with a relative abundance < 1%. (B) Differential abundance of bacterial species associated with asymptomatic and PRR-symptomatic avocado trees according to the Wald test. Values of Log2FoldChange ≠ 0 and *P* < 0.05 are considered as differentially abundant in each condition (n = 3 mRNA pools for each tree condition).

*Coxiella burnetti, Legionella pneumophila, Candidatus* Solibacter, *Gemmatimonas phototrophica, Gemmatimonas aurantiaca*, and *Streptomyces* sp. (Fig. 4A) were amongst the most active rhizosphere bacterial taxa in both conditions. A differential expression analysis showed that *Mycoplasma haemofelis* (Log2FC = -7.26), *Anaeromyxobacter* sp. (Log2FC = -5.59), *Chroococcaceae* (Log2FC = -5.09), *Pilibacter termitis* (Log2FC = -5.09), and *Rickettsia rhipicephali* (Log2FC = -4.85) were the most significantly enriched bacterial genera in the rhizosphere of asymptomatic avocado trees. On the other hand, *Burkholderia* sp. (Log2FC = 7.26), *Hydrogenophaga* sp. (Log2FC = 7.18), *Rhodanobacter* sp. (Log2FC = 6.92), *Mycoplasma orale* (Log2FC = 6.81), *Modestobacter* sp. (Log2FC = 6.75), *Rhizobium* sp. (Log2FC = 6.65), and *Arthrobacter* sp. (Log2FC = 6.57) were significantly enriched in the rhizosphere of PRR-symptomatic avocado trees (Fig. 4B).

**Figure 5:**
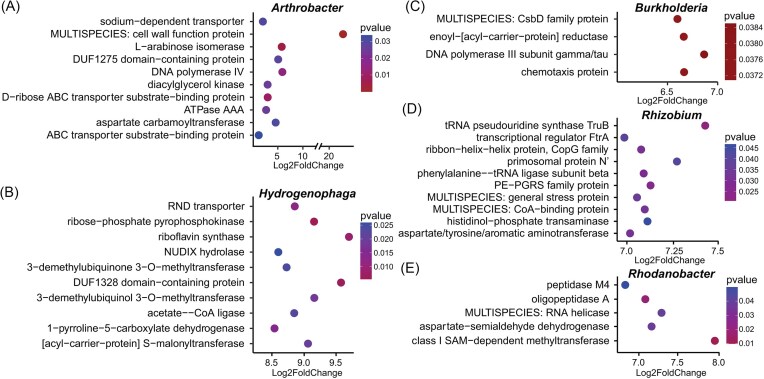
Functional profile of the most abundant and active bacterial genera in the rhizosphere of PRR-symptomatic avocado trees. (A)—(E) Each panel enlists the top functions for each bacterium with Log2FoldChange values > 0 (enriched in the PRR-symptomatic condition, as compared with the asymptomatic condition) and a *P*-value < 0.05 (n = 3 mRNA pools for each tree condition).

The functions performed by the enriched genera in the PRR-symptomatic avocado trees were further analyzed (Supplementary Table S2). In accordance with results obtained in the functional profiling of the whole active community, *Burkholderia* sp., *Hydrogenophaga* sp., *Rhodanobacter* sp., *Rhizobium* sp., and *Arthrobacter* sp., the most active taxa from the PRR-symptomatic trees, expressed genes associated with stressful conditions and symbiotic processes, the biological functions pathogenesis and virulence, protein activation and nutrient acquisition, cell signaling, and bacterial and plant ion-homeostasis. Primary metabolism in these bacterial taxa was represented by genes encoding peptidases, transporters, kinases, and hydrolases (Fig. 5), which were only enriched in the PRR-symptomatic condition.

## Discussion

*Phytophthora cinnamomi* is one of the most devastating pathogens infecting avocado trees. To better understand the impact of this pathogen on the avocado rhizosphere bacteriome, a metabarcoding strategy was combined with a metatranscriptomic approach to determine the compositional and functional shifts in avocado rhizosphere bacterial communities associated with PRR under field conditions. The impact of *P. cinnamomi* on the active bacteriome in the avocado rhizosphere was investigated here for the first time. Results showed that the enrichment of some bacterial genera in the PRR-symptomatic avocado trees might be related to the release of resources by root rot and that the active bacteria could be taking advantage of these released nutrients, as the metatranscriptomic analysis highlighted an activated carbohydrate metabolism in the rhizosphere of PRR-symptomatic trees. These results confirmed the first hypothesis regarding the enrichment of copiotrophic taxa in the rhizosphere of infected trees. Moreover, the second hypothesis was confirmed since results showed that the most active taxa in the rhizosphere of PRR-symptomatic trees expressed genes associated with a stress response, as the enrichment of several transporters associated with stress responses was detected. Some of these active taxa are known plant growth promoters and pathogen antagonists, which suggests they may have been recruited by the infected host as part of its defense response. By pinpointing such potential antagonists, this study aimed to contribute to the eventual development of microbiome-based strategies that could be included into the integrated management of PRR in avocado. As these findings were based on a single-event, single-location sampling, further replicated field studies should aim at confirming the identification and validation of such beneficial microorganisms.

The effect of soil-borne pathogens on the diversity of rhizosphere bacterial communities has been shown to vary, ranging from increases (Yang et al. 2001, Mendes et al. 2023) to decreases in rhizobacterial diversity in infected plants (Wei et al. 2018, Zhou et al. 2021), or with no effects (Hassani et al. 2023, Reverchon et al. 2023; Yin et al. 2021). Here, *P. cinnamomi* did not affect alpha diversity metrics nor community structure (beta diversity) of the avocado rhizobacterial community, consistent with the findings of Solís-García et al. (2021) in Veracruz (Mexico) orchards. Root rot symptoms in general have been shown to produce stronger alterations in fungal than bacterial communities in the rhizosphere (Jiang et al. 2019, Díaz-Cruz and Cassone 2022, Muñoz-Castellanos et al. 2024), which is attributed to nutrient and niche competition. As observed in other pathosystems, changes in diversity metrics may become more evident when pathogen infection induces an increased resource competition between the pathogen and the rhizosphere microbiome, e.g. between *Ralstonia solanacearum* and rhizobacteria in tobacco (Wang et al. 2022) or *Fusarium* and fungi in the sugarcane rhizosphere (Li et al. 2022). While bacteria may favor labile C sources (Wang and Kuzyakov 2024), *P. cinnamomi*, a soil-borne necrotrophic oomycete, displays an arsenal of glycoside hydrolases for the direct or indirect break-down of plant cell walls and plant cells, to feed on dead tissue (Bradley et al. 2022). These reports suggest a potentially stronger effect of *P. cinnamomi* on fungal than bacterial communities in the avocado rhizosphere and highlight the need for further study of inter-microbial nutrient competition in the rhizosphere of infected trees.

### Changes in the abundance of certain bacterial genera may be related to energy resources

Although *P. cinnamomi* did not significantly alter the structure and diversity of bacterial communities in the avocado rhizosphere, compositional shifts were detected in PRR-symptomatic trees compared to asymptomatic trees. Given that we did not find significant changes in soil chemical characteristics between the tree conditions, these shifts in composition were likely induced by the presence of the pathogen. The lack of differences in alpha-diversity or beta-diversity metrics between rhizosphere bacterial communities associated with asymptomatic and symptomatic trees has been previously described in avocado as well as other crops, such as pecan trees (Solís-García et al. 2021, Muñoz-Castellanos et al. 2024). Consistent with our findings, these authors reported that, despite the lack of effects of the disease on bacterial diversity metrics, root rot in avocado or pecan trees induces compositional changes in bacterial communities. They argue that intra- and inter-replicate variability (Muñoz-Castellanos et al. 2024) as well as larger data dispersion in the symptomatic condition (Solís-García et al. 2021) could have obscured possible differences in terms of community structure between tree conditions, as may have occurred in the present study. Here, a high proportion of the detected ASVs that were exclusive of symptomatic or asymptomatic trees were rare taxa (relative abundance ≤ 0.005). Rare microbial taxa are fundamental for plant health and key for their host´s response to biotic and abiotic stressors (Hol et al. 2010, Jousset et al. 2017, Bejarano-Bolívar et al. 2021). However, they may also be highly sensitive to environmental perturbations, which may explain the turnover of exclusive taxa in the rhizosphere of asymptomatic and PRR-symptomatic trees (Xu et al. 2021, Sun et al. 2022).

Enriched bacterial taxa in the rhizosphere of asymptomatic avocado trees included *Variovorax, Rhodanobacter, Anaeromyxobacter, Candidatus* Udaeobacter, and *Chryseolinea*. The *Variovorax* genus is reported to promote plant growth, manipulating *Arabidopsis thaliana* hormones to balance the effects of a synthetic community of bacteria on root growth (Finkel et al. 2020) or enhancing wheat germination under salt stress conditions (Acuña et al. 2024). Moreover, *Variovorax* is also associated with root rot tolerance in wheat (Yin et al. 2021). Wang et al. (2022) found that *Variovorax* is more abundant in healthy roots of *Panax notoginseng* than in plants with symptoms of root rot and suggested that this genus could be a promising biological control agent against *P. notoginseng* root rot. Similarly, Pereira et al. (2025) reported that *Variovorax paradoxus* exhibits *in vitro* antagonistic activity against *F. oxysporum*. On the other hand, the enrichment of *Rhodanobacter* in the ginseng rhizosphere is shown to increase its resistance to disease, improving crop yield and quality (Li et al. 2024). Moreover, novel strains of *Rhodanobacter* have displayed antagonistic activities *in vitro* against the fungal pathogens *Cylindrocladium spathiphylli* (De Clercq et al. 2006) and *Fusarium solani* (Huo et al. 2018). Overall, these studies suggest that the presence of these bacteria in the rhizosphere of asymptomatic avocado trees may contribute to maintaining the health of their host. Alternatively, the enriched taxa in the rhizosphere of asymptomatic avocado trees may be more susceptible to the presence of the pathogen than other rhizosphere bacterial taxa (Chavarro-Carrero et al. 2024), although previous reports of antagonistic activity displayed by *Variovorax* or *Rhodanobacter* against soil-borne pathogens support a possible contribution to plant defense. Regarding *Chryseolinea* and its interactions with plant pathogens, results from the literature are contrasting. Ou et al. (2019) found that *Chryseolinea* may play a role against *F. oxysporum* establishment in disease suppressive soils, while Tang et al. (2023) describe a positive correlation of this taxon with *Fusarium* wilt incidence; further studies are thus necessary to elucidate whether *Chryseolinea* could assist trees in mitigating *P. cinnamomi* infection. Although little studied, this genus, as a member of the Cytophagales, has the capability to produce cellulolytic and chitinolytic enzymes, making it a suitable candidate to antagonize the growth of soil-borne fungal and oomycete pathogens (Glavina et al. 2010, Tan et al. 2023).

Conversely, the significant increase in ASVs from the genera MND1, RB41, and *Nitrospira* in both PRR-symptomatic and asymptomatic rhizosphere soil samples could respond to different mechanisms between conditions. Wei et al. (2024) reported the enrichment of these three genera in rhizosphere soils from healthy tobacco plants compared to plants infected by *R. solanacearum* and suggest that these taxa may be related to plant-root symbiosis and nutrient acquisition, nitrogen cycling, and organic matter decomposition. Moreover, they found that RB41 and MND1 positively correlate with seven active metabolites involved in the synthesis of antibiotics and alkaloids (Wei et al. 2024), which may indicate their role in disease suppression. MND1 has also been described as being more abundant in the rhizosphere of healthy plants compared to plants infected by *R. solanacearum* (Wen et al. 2020). On the other hand, members of the genus *Nitrospira* have been described as complete ammonia oxidizers in forest, grassland, and agricultural soils (Hu et al. 2021), thus playing an important role in soil nitrification. *Nitrospira* was also found to be negatively related to disease incidence in tobacco, putatively due to its contribution to nitrate production and plant disease resistance (Li et al. 2025). Collectively, these results suggest that the enrichment of MND1, RB41, and *Nitrospira* in the rhizosphere of PRR-symptomatic trees may be due (1) the release of resources due to root rot and (2) their recruitment as part of the plant “cry-for-help”, as possible contributors to plant health and defense response (Mur et al. 2016, Sun et al. 2021). Downstream experimental validation is warranted to elucidate whether enriched taxa in the rhizosphere of infected trees result from an increase in available carbohydrates or from a plant defense response, as our study constitutes a single time-point comparison of bacterial communities associated with asymptomatic and PRR-symptomatic trees. To narrow these future experiments, further functional analyses were performed to dig deeper into the saprotrophic activity and/or potential of these bacterial taxa for disease mitigation.

### Rhizobacteria may be responding to the release of resources by *Phytophthora cinnamomi* root-rot

The functional enrichment analysis based on COG and KEGG databases showed that the active bacterial community changed depending on the health status of the host tree. More enriched pathways were found in the mRNA pools from the rhizosphere of PRR-symptomatic trees than in those from asymptomatic avocado trees. The enriched metabolic routes in infected trees were generally related to carbohydrates metabolism, nitrogen metabolism, and to post-transcriptional and translation regulation.

The reiterative enrichment of the aconitate hydratase pathway in mRNA pools from infected trees in both COG and KEGG annotations suggested that carbohydrate metabolism was stimulated in the rhizobacterial communities associated with PRR-symptomatic trees. This enzyme participates in the tricarboxylic acid (TCA) cycle, catalyzing the reversible hydration of cis-aconitate to produce citrate or isocitrate. Additionally, the enrichment of the aconitate hydratase in symptomatic trees combined with that of 2-methylisocitrate dehydratase confirmed that the 2-MC cycle was occurring too. Both the TCA and 2-MC cycles are coupled, since they share enzymes such as the aconitate hydratase and the 2-MC cycle provides substrates for the TCA cycle. The significant enrichment of the 4iron-4sulfur cluster (COG), another key player in carbon metabolism, and of at least ten more transcripts related to the carbohydrate metabolism in the mRNA pools from the rhizosphere of infected trees, indicated that active rhizosphere bacteria in symptomatic avocado trees were likely taking advantage of the C resources released by root rot. These findings are consistent with those by Shu et al. (2019), who detected with metagenomics an increase in genes related to carbohydrate metabolism in the rhizosphere of avocado trees infected by *P. cinnamomi* and suggest that the presence of the pathogen stimulates the primary metabolism of the rhizosphere bacterial community.

On the other hand, transcripts related to maintenance of the cellular proteostasis network, which allows bacteria to acclimatize to abiotic stress, were enriched in the asymptomatic condition. The enrichment of chaperone protein families, such as Hsp20 and protein folding chaperone confirmed the role of the rhizobiome in providing stress tolerance to the host plant; the heat shock protein Hsp20 has been reported to increase following exposure to stress, including temperature changes (Xue et al. 2019) or desiccation resistance (León-Sobrino et al. 2024). Thioredoxin systems (*trxA* and *trxB*) act as a sensor, favoring stress adaptation, principally to reactive oxygen species (ROS), O_2_, and reactive N species. These protein systems control the modifications of oxidized proteins (thiol group), ensuring protein repair and maintaining the thiol homeostasis in the cell (Anjou et al. 2024). FtsH and Clp proteases belong to the Hsp100 family proteases. Clp proteins are involved in stress response, sporulation, swarming, motility, and biofilm formation, whereas FtsH metalloprotease is involved in sporulation initiation, biofilm formation, cell envelope stress, heat shock and is directly involved in protein quality control through the degradation of misfolded or damaged proteins (Harwood and Kikuchi 2021). Overall, the depletion of these transcripts in the mRNA pools from the PRR-symptomatic avocado trees could indicate a loss of resilience in the event of additional stressors, such as drought or nutrient scarcity.

### The dominant active bacteria in symptomatic avocado trees are associated with plant growth-promotion

The active bacterial communities in the rhizosphere of PRR-symptomatic avocado trees were dominated by *Burkholderia, Hydrogenophaga, Rhodanobacter, Modestobacter, Rhizobium*, and *Arthrobacter*. Most of these taxa have been associated with plant growth promotion and disease suppression, which suggests they could have been recruited by the plant upon infection to assist its defense response. For example, *Burkholderia* and *Arthrobacter* strains have been isolated from the sugarcane rhizosphere and displayed antagonistic activity against *Fusarium commune* GXUF-3, suppressing its mycelial growth and spore germination (Li et al. 2022). *Arthrobacter* has also been reported for its anti-oomycete and plant growth-promoting activities in several plant species (Velázquez-Becerra et al. 2013, Town et al. 2016, Méndez-Bravo et al. 2018). However, it is also possible that these taxa were attracted by the nutrient release following root necrosis. For example, *Rhodanobacter*, which was enriched in the rhizosphere of asymptomatic avocado trees (metabarcoding), was detected as more active in the rhizosphere of infected trees (metatranscriptomics), with an increased expression of peptidase M4 and oligopeptidase (Hasan et al. 2021). This may indicate an increase in resource acquisition in a nutrient-rich condition.

However, some of the enriched transcripts in these bacterial taxa point at a possible contribution to PRR mitigation. One of the differentially expressed transcripts associated with *Burkholderia* in the present study was chemotaxis protein. Bacteria use chemotaxis to sense changes in chemical concentration gradients in the environment, allowing them to search for food, avoid toxins, and respond to changing environmental conditions (Wadhams and Armitage 2004). *Burkholderia* spp. have been reported to display a positive chemotactic response to the root exudates of *Panax notoginseng* in autotoxic ginsenoside stress, which allows them to enhance their root colonization and protect the plant from root rot disease (Deng et al. 2023). In the rhizosphere of PRR-symptomatic avocado trees, *Arthrobacter* was actively transporting substrates from the surrounding environment, as the relative abundance of transcripts related to ABC transporters (D-ribose, substrate-binding protein) increased for this genus. ABC transporters are involved in the import and export of a wide range of substrates, such as sugars, amino acids, ions and complex organic molecules (Tanaka et al. 2018), supporting the hypothesis that *Arthrobacter* may have benefited from the release of carbohydrates following root rot (Alfaro-García et al. 2026). However, D-ribose has also been reported, among other carbohydrates, to stimulate the growth and diversity of tomato rhizobacteria, resulting in a reduction of the pathogen *R. solanacearum* abundance (Wen et al. 2023). D-ribose production occurs through the pentose phosphate pathway (PPP), which has an important role in maintaining redox homeostasis and activating stress responsive gene expression under oxidative stress (Gupta and Gupta 2021). As H_2_O_2_ has been described to increase in the interaction of avocado-*P. cinnamomi* (García-Pineda et al. 2010), the activation of PPP could help rhizobacteria to deal with the ROS produced by the avocado during infection. Although stress responses were less represented in the global expression profile of the mRNA pools from PRR-symptomatic trees than in those from asymptomatic trees, possibly indicating a loss of resilience of rhizosphere bacterial communities upon tree infection, the presence of genes belonging to the category of stress response expressed by the most active bacterial genera in infected trees suggest that these most active taxa kept the ability to respond to adverse conditions.

## Conclusions and final considerations

The present study confirmed that *P. cinnamomi* altered the avocado rhizobacterial community at the compositional and functional levels. The active bacterial community associated with PRR-symptomatic avocado trees displayed an induced gene repertoire ranging from an enhanced carbohydrate metabolism to the expression of genes related to stress response, cell signaling, ion-homeostasis, virulence factors, and symbiotic interactions with plants. These findings highlight the importance of combining compositional and functional microbiome analyses to distinguish between beneficial microbial taxa potentially contributing to disease mitigation and those merely attracted by the release of labile C compounds following root rot. Future studies will aim at complementing our understanding of the avocado microbiome shifts induced by *P. cinnamomi* by investigating the effect of the pathogen on rhizosphere fungal communities and on root exudation patterns through metabolomics. Furthermore, a complementary time-course infection experiment will provide a deeper understanding of the effects of *P. cinnamomi* on the avocado rhizosphere microbiome throughout the disease progression (Reverchon et al. 2023). Collectively, this multi-omics study of the avocado-*P. cinnamomi* pathosystem contributes to identify potential biocontrol bacterial taxa, which bioactivity should be validated in downstream experiments. Confirming their recruitment by the plant and their antagonistic activity against *P. cinnamomi* would assist in establishing the bases for future microbiome manipulation strategies and for the build-up of disease-suppressive soils that could help mitigate the impact of this important soil-borne pathogen.

## Supplementary Material

xtag025_Supplemental_Files
