# Supplementary material for: Phytophthora root rot induces compositional and functional changes in avocado rhizosphere bacterial communities
Source: FEMS Microbes. 2026 May 12;7:xtag025. doi: 10.1093/femsmc/xtag025 (PMC13220768; doi:10.1093/femsmc/xtag025)
Supplement: xtag025_Supplemental_Files [file xtag025_supplemental_files.zip › Supplementary material - MS - 2025 - Alfaro-Garcia-R1.docx]

**Supplementary Material**

***Phytophthora* root rot induces compositional and functional changes in avocado rhizosphere bacterial communities**

Rosaura G. Alfaro-García^1,2^, Pablo Vargas-Mejía^3^, Violeta Patiño-Conde^2^, Eria A. Rebollar^4^, José A. Guerrero-Analco^5^, Julio Vega-Arreguín^3^, Frédérique Reverchon^1^*, Alfonso Méndez-Bravo^2,6^*

1. Red de Diversidad Biológica del Occidente Mexicano, Instituto de Ecología, A. C., Centro Regional del Bajío. Av. Lázaro Cárdenas 253, Centro. 61600 Pátzcuaro, México.
2. Laboratorio Nacional de Análisis y Síntesis Ecológica, Escuela Nacional de Estudios Superiores, Unidad Morelia, Universidad Nacional Autónoma de México, Antigua carretera a Pátzcuaro 8701, Ex Hacienda de San José de la Huerta. 58190 Morelia, México.
3. Laboratorio de Ciencias Agrogenómicas and Laboratorio Nacional PlanTECC, Universidad Nacional Autónoma de México. Blvd. UNAM 2011, Predio El Saucillo y Comunidad Los Tepetates, El Potrero. 37684 León, México.
4. Centro de Ciencias Genómicas, Universidad Nacional Autónoma de México. Av. Universidad s/n, Universidad Autónoma del Estado de Morelos. 62210 Cuernavaca, México.
5. Red de Estudios Moleculares Avanzados, Instituto de Ecología, A.C. Carretera Antigua a Coatepec 351, El Haya. 91073 Xalapa, México.
6. SECIHTI, Ciudad de México, México.
7.
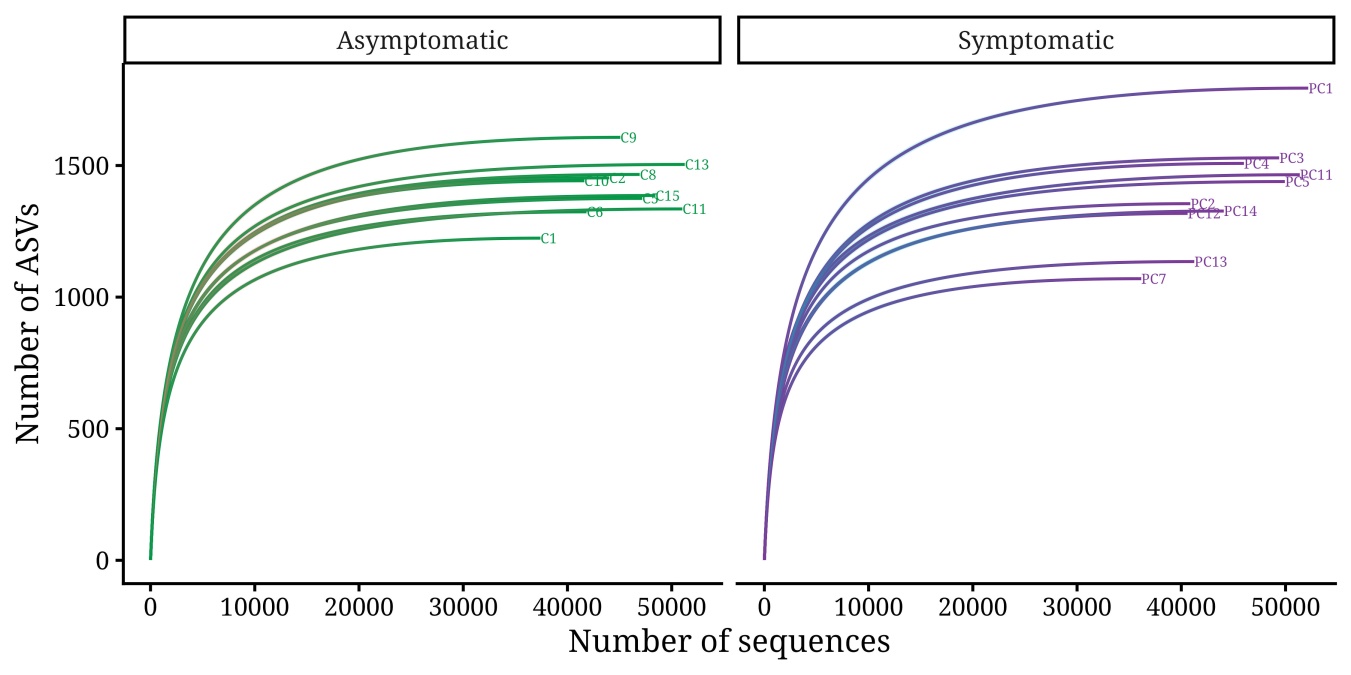


Supplementary Figure 1. Rarefaction curves of the observed bacterial ASVs associated with asymptomatic and PRR-symptomatic avocado trees.


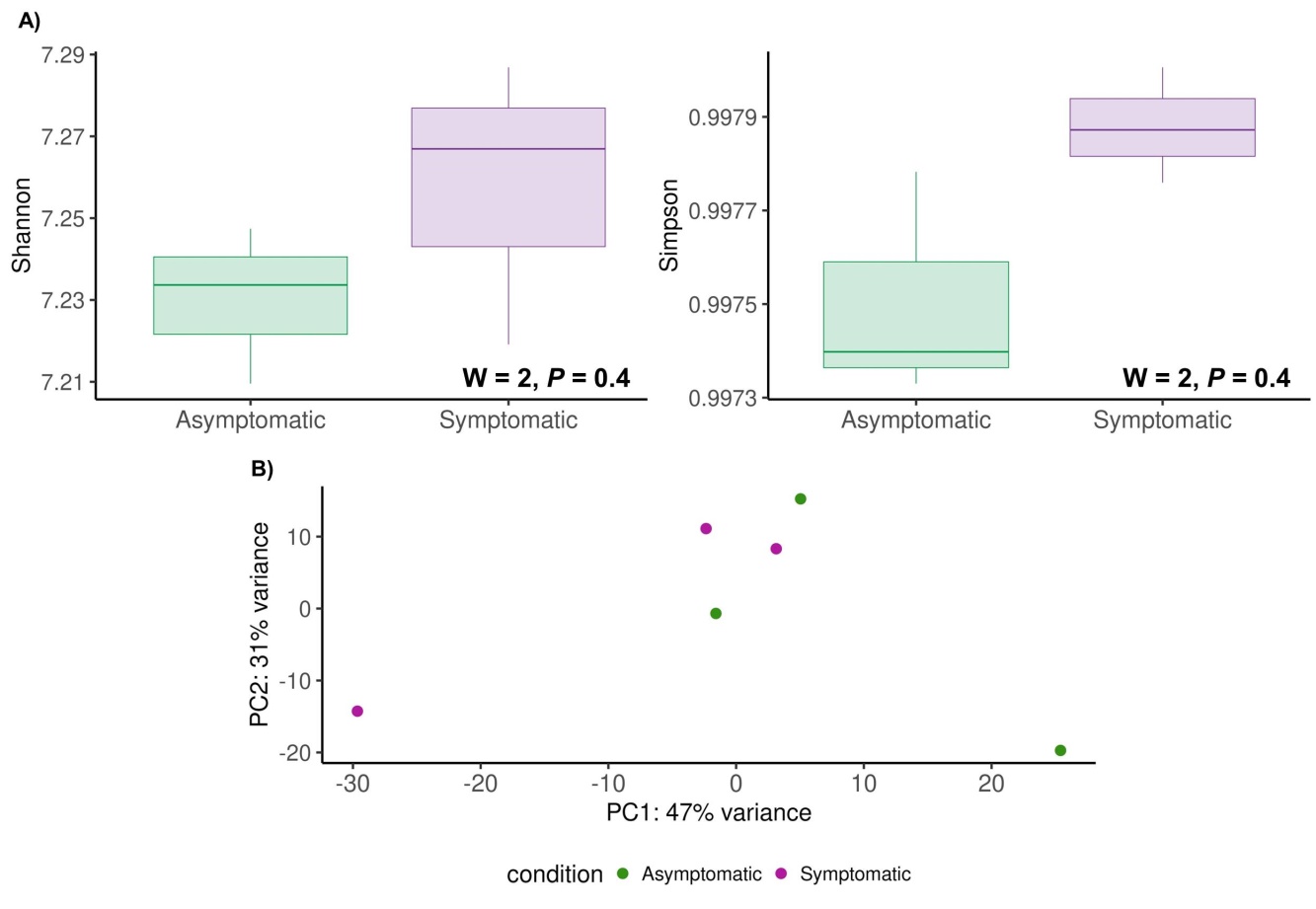


Supplementary Figure 2. Taxonomic diversity and structure of the active bacterial community in the rhizosphere of asymptomatic and PRR-symptomatic avocado trees. A) Shannon and Simpson indices of active rhizobacterial communities associated with asymptomatic and PRR-symptomatic avocado trees, as inferred from the metatranscriptome. The W and *P* values were calculated with the Mann-Whitney-Wilcoxon test. B) Principal component analysis (PCA) showing the functional structure of asymptomatic and PRR-symptomatic avocado trees retrieved from expression data across all the metatranscriptome (three RNA sequencing libraries per tree condition).

Supplementary Table S1. Significantly expressed genes by the active bacterial community in the rhizosphere of asymptomatic and PRR-symptomatic avocado trees.

Supplementary Table S2. Significantly expressed genes by the most active genera in the rhizosphere of PRR-symptomatic avocado trees.
