# Supplementary material for: Phytophthora root rot induces compositional and functional changes in avocado rhizosphere bacterial communities
Source: FEMS Microbes. 2026 May 12;7:xtag025. doi: 10.1093/femsmc/xtag025 (PMC13220768; doi:10.1093/femsmc/xtag025)
Supplement: xtag025_Supplemental_Files [file xtag025_supplemental_files.zip › FEMSMC-2025-067.R2 supplementary data - reviews and author response.docx]

**Reviews of manuscript FEMSMC-2025-067**

**Reviewer 1: Victor Flores Nuñez, Albrechts University**

**Recommendation: Major Revision**

Phytophthora root rot induces compositional and functional changes in avocado rhizosphere bacterial communities

The authors assessed the taxonomic and functional diversity of the rhizospheric bacterial communities in avocado trees, particularly focusing on the differences between trees with symptoms of Phytophthora root rot (PRR) and trees without symptoms of disease. The relevance of the work is focused on the economic value of avocado in Mexico. It seems that the study was hypothesis-free with the aim of identifying potential bacterial taxa and bacterial traits associated with the disease and its mitigation. The authors found evidence of a functional enrichment of carbon metabolism and stress response consistent with the cry for help model.

I found it extremely valuable that the authors approached this work using meta-transcriptomics to determine the active rhizospheric bacteria and their functions during the disease. I agree that studying how pathogens modify the microbiome of their hosts is a key subject to generate strategies to manage plant diseases.

In general, I found the background, strategy, and results appropriately described and easy to follow. The figures have good quality too. However, some elements in the results and discussion might benefit from extra clarification.

Introduction:

The authors didn't explicitly mention a hypothesis; however, they continuously refer to known microbial processes such as dysbiosis (a disbalance in the microbiota that directly affects plant health) and the cry-for-help hypothesis (plants recruiting protective microorganisms). This makes me wonder what the authors’ expectation or hypothesis was in the context of their scientific question, as these two scenarios describe quite opposite processes. Furthermore, there are other mechanisms described in the literature that explain how pathogens modify the microbiota of their host (e.g., secretion of effector proteins, triggering of the plant immune response, etc.). I believe that a hypothesis is also important to guide the reader through the strategy, comparisons, and discussion.

Methods:

L116: Is this soil rhizospheric or bulk?

L188: I suggest including how the Venn diagrams were calculated. What were the abundance and prevalence thresholds to determine if an ASV is present within a group?

R and packages should have citations.

SRA number does not give hits in NCBI

Results:

L260: I found the Venn diagram analysis difficult to interpret, especially without describing how it was calculated. Only 3.8% ASVs are shared, which indicates that the majority of ASV are unique to each condition. This makes it seem that these communities are very different; however, there is no significant difference in their diversity and structure. Also, this finding was not discussed further; therefore, I would reconsider its relevance in the work.

L274: This section describes the diversity analysis of the meta-transcriptomic approach; however, there is no description in the methods about this. Also, Supplementary Figure 2 shows only 6 samples analyzed, while the methods described 19 samples processed. It is not clear whether this figure represents the transcript diversity or the taxonomic diversity inferred from the transcriptome.

L311-317: The high abundance of Mycoplasma haemophelis transcripts, which is an obligate parasite of cats, is puzzling. Does your annotation strategy really allow you to classify these genes to a bacterial species? Or is it a misinterpretation?. Would the following be a better description of these results? --The most significantly enriched transcripts were homologous to genes from Mycoplasma haemofelis?--. If not, would you consider Mycoplasma haemophelis as rhizospheric or plant-associated?

L324-330: The authors describe the functions performed by the most enriched bacterial genera in the PRR-symptomatic trees; however, I consider that these findings lack a point of comparison. Are there any genes in these categories also enriched in the asymptomatic trees? Are these genes associated with the same genera in asymptomatic trees? What are the functions of the genera enriched in asymptomatic trees? My intention is not for the authors to answer these questions but for them to better frame these findings in the context of a hypothesis.

Discussion:

L344-346. It would be great to have more arguments or examples on how these findings could contribute to the development of microbiome-based strategies for plant protection.

L350-362. The authors argue that the differences in substrate preference by bacteria and pathogens could explain the absence of changes in community diversity in the diseased trees. However, these arguments might be ignoring the space these microorganisms are occupying, the bacteria in the rhizospheric soil particles, and the pathogen in the root (surface and vascular tissues?). Would resource competition apply under these circumstances?.

L353: The bacterial diversity and structure were not significantly different between symptomatic and asymptomatic trees. In my opinion, this does not necessarily mean that there is a “lack of effect” of P. cinnamomi (L353), because the authors are not evaluating the infection process alone (e.g., a controlled infection experiment) but already diseased trees (in the context of their environment). It seems that the avocado bacterial rhizospheric communities are resilient toward the disease, rather than the pathogen not having an effect on diversity and structure.

L362-363: I don't agree that the findings suggest this. This line is based on the author's argumentation of resource competition, not based on the actual findings. I suggest rephrasing this part

L373: The authors argue that the enriched ASV from asymptomatic trees might be contributing to maintaining the host health based on similar taxa in other pathosystems. While I agree, I think this should be toned down, because these taxa are not absent from symptomatic trees (just less abundant) and might be contributing to plant health there too. Also, there are alternative explanations. Would you consider that these taxa are actually the most sensitive to the PRR disease because they have lower abundance in the symptomatic trees compared to asymptomatic trees? Filamentous pathogens are known to secrete antibacterial effectors with different degrees of specificity, which might explain this pattern.

L436. The transcriptomic findings suggest that the bacterial communities are reacting to Carbon released from the roots. This is based on the enrichment of 3 proteins: from the TCA, 2-MC and the 4irosn-4sulfur domain. I wonder if this is enough evidence to support one of the main conclusions of this work. Can the authors show evidence that these genes are co-expressed with other important genes for carbon metabolism? Is there experimental evidence about the differences in carbon composition in the rhizosphere of these plants under disease?

There seems to be a small disconnect regarding stress-response results and discussion. The abstract indicates that the most active genera in PRR-symptomatic trees have transcripts of stress response and signaling (L37), while the results only indicate that they had transcripts associated with stress conditions (L326), but it is unclear which ones in Figure 5. Moreover, the discussion mostly highlights the stress genes that are enriched in the asymptomatic trees and depleted in PRR trees, indicating a loss of resilience (L454-456). The authors do not really discuss the results in L37 and L326 that seem to be part of the main findings; they mostly discuss other microbial processes like carbon metabolism and chemotaxis in page 21 and 22. Do these findings indicate that the stress response is important in both conditions?

**Reviewer 2**

**Recommendation: Major Revision**

You present a substantial multi-omics dataset, and the metabarcoding and metatranscriptomic workflows appear carefully executed. However, the manuscript has a weakness based around the study design and what I believe is an overreach in interpreting those results. You frame the results as evidence for plant-driven recruitment (“cry-for-help”) and microbiome-mediated mitigation, yet the work uses a single cross-sectional sampling event in one orchard, with no manipulation and no tracking of change through time. A revised manuscript needs tighter alignment between claims, design, and statistics (less over reaching)

1. You interpret symptomatic versus asymptomatic differences as active recruitment and mitigation. A single time point observational comparison does not test recruitment, directionality, or mitigation. It only supports association. Please either add experimental evidence (time series across disease progression, inoculation, exudate manipulation, targeted functional assays), or reframe the manuscript explicitly as a descriptive association study and remove mechanistic language from the abstract, results interpretation, and conclusion.

2. The manuscript repeatedly presents recruitment as a demonstrated outcome. An alternative explanation fits the design better, namely resource release and altered chemistry after root damage or necrosis, which would favour opportunistic taxa without requiring plant-driven recruitment. Please treat recruitment as a hypothesis with clear predictions, and rewrite sections that currently state recruitment or mitigation as findings.

3. With one orchard and one sampling date, spatial structure and management variation pose major confounding risks. Symptomatic and asymptomatic trees might differ in soil, irrigation, microtopography, rootstock, cultivar, age, or prior management. At present, the manuscript does not convince the reader that PRR status explains most of the observed differences.

Please include a clear description of orchard layout and sampling strategy, including how you matched symptomatic and asymptomatic trees. I would expand the soil characterisation to represent all sampled trees, not a small subset and then test to see if the soil characteristics observed are diving aby of the changes ie. Incorporate soil covariates and spatial structure into the community analyses, and report how these factors shift conclusions.

4. Pooling samples into a small number of libraries reduces effective replication and masks between-tree variability. I understand why, it is still expensive and you may not have had enough RNA for the sequencing. Please state the effective sample size for transcriptomics clearly and carry that through the analysis and wording. Interpret gene expression patterns as pool-level signals rather than tree-level (individual) biology.

5. You report non-significant outcomes for key community-level tests, yet you use strong dysbiosis language in the interpretation and conclusion. Please align the conclusions with the statistics. If the strongest signal sits in differential abundance of specific taxa or pathways rather than whole-community separation, then focus your conclusions there and quantify effect sizes and uncertainty.

6. The title and abstract currently promise mechanistic insight and yet the neither the sampling design nor the findings support this. The conclusion reads as confirmation of recruitment and mitigation.

7. Improve resolution and font sizes, and ensure legends allow the reader to interpret the figure without referring back to the main text. Figure 1 in particular is very hard to read

There have - i believe been quite a few microbiome studies looking at PRR - you have selected a small number to reference - the references dont have to only be for avocado if they are relavent.

Farooq, Q.U.A., McComb, J., Hardy, G.E.S.J. and Burgess, T.I., 2024. Soil amendments for management of Phytophthora root rot in avocado and their impact on the soil microbiome. Journal of Plant Pathology, 106(2), pp.439-455.

Reverchon, F., García-Meléndez, M., Guevara-Avendaño, E., Mora-Chávez, O., Solís-García, I.A., Dáttilo, W., Guererro-Analco, J.A., Méndez-Bravo, A., Monribot-Villanueva, J.L., Patiño-Conde, V. and Pineda-García, F., 2023. Shifts in the rhizosphere microbiome and exudation profile of avocado (Persea americana Mill.) during infection by Phytophthora cinnamomi and in presence of a biocontrol bacterial strain. CABI Agriculture and Bioscience, 4(1), p.23.

Balbín-Suárez, A., Jacquiod, S., Rohr, A.D., Liu, B., Flachowsky, H., Winkelmann, T., Beerhues, L., Nesme, J., J. Sørensen, S., Vetterlein, D. and Smalla, K., 2021. Root exposure to apple replant disease soil triggers local defense response and rhizoplane microbiome dysbiosis. FEMS microbiology ecology, 97(4), p.fiab031.

Giachero, M.L., Declerck, S. and Marquez, N., 2022. Phytophthora root rot: Importance of the disease, current and novel methods of control. Agronomy, 12(3), p.610.

**Associate Editor Recommendation: Major Revision**

**EIC Decision: Major Revision**

AE felt it was borderline reject - Major revision. I decided to go with major revision but stated in the letter that they must address Reviewer 2 comments thoroughly as it can still be rejected after revision.

**Author's Response to Decision Letter for (FEMSMC-2025-067)**

Dear Dr. Mendez-Bravo,

Thank you for submitting the above manuscript to our journal. It has now been expertly refereed and recommendations are given below.

You see below that the reviewers, although they found merit in your study, have raised a number of concerns. Although all of the comments must be addressed, please pay particular attention to reviewer 2 comments. Specifically to point 1, 5 and 6. These require an extensive revision and refocusing of the manuscript or additional data to justify the conclusions. Furthermore the discussion should consider limitations of the work. These issues are central to the manuscript and must be fully addressed prior to acceptance for publication.

If you are able to address these comments and the points I mention below, then I will be happy to consider a revised manuscript. Please remember to also submit a version of the manuscript showing all the changes to facilitate the review.

Please return your revised manuscript by 24-Mar-2026 (EDT).

R: We thank the Editor for the opportunity to resubmit our work to FEMS Microbes. All comments made by the reviewers have been addressed and the manuscript has been substantially restructured. In particular, we have tuned down our conclusions and emphasized the limitations of our work regarding the importance of carrying out more samplings. We added methodological details where requested and have improved figure resolution. We included hypotheses as suggested and added more discussion regarding the numbers of transcripts enriched in the symptomatic condition and the importance of saprotrophy as a result of root necrosis, in the context of other root rot studies. We believe these modifications greatly improved the clarity and coherence of our manuscript.

Reviewer 1

The authors assessed the taxonomic and functional diversity of the rhizospheric bacterial communities in avocado trees, particularly focusing on the differences between trees with symptoms of Phytophthora root rot (PRR) and trees without symptoms of disease. The relevance of the work is focused on the economic value of avocado in Mexico. It seems that the study was hypothesis-free with the aim of identifying potential bacterial taxa and bacterial traits associated with the disease and its mitigation. The authors found evidence of a functional enrichment of carbon metabolism and stress response consistent with the cry for help model.

I found it extremely valuable that the authors approached this work using meta-transcriptomics to determine the active rhizospheric bacteria and their functions during the disease. I agree that studying how pathogens modify the microbiome of their hosts is a key subject to generate strategies to manage plant diseases.

R: Thank you for your encouraging comments and careful revision of our manuscript.

In general, I found the background, strategy, and results appropriately described and easy to follow. The figures have good quality too. However, some elements in the results and discussion might benefit from extra clarification.

Introduction:

The authors didn't explicitly mention a hypothesis; however, they continuously refer to known microbial processes such as dysbiosis (a disbalance in the microbiota that directly affects plant health) and the cry-for-help hypothesis (plants recruiting protective microorganisms). This makes me wonder what the authors’ expectation or hypothesis was in the context of their scientific question, as these two scenarios describe quite opposite processes. Furthermore, there are other mechanisms described in the literature that explain how pathogens modify the microbiota of their host (e.g., secretion of effector proteins, triggering of the plant immune response, etc.). I believe that a hypothesis is also important to guide the reader through the strategy, comparisons, and discussion.

R: Hypotheses are now included in the last paragraph of our Introduction. We also added a paragraph in the Introduction (L64, clean version of the revised manuscript) to better describe the mechanisms leading to shifts in rhizosphere bacterial communities induced by soil-borne pathogens and sustain such hypotheses.

Please refer to the revised manuscript:

L114, clean manuscript: “Based on findings from previous studies investigating the effects of root rot on rhizosphere microbiomes (Magagula et al., 2025; Shu et al., 2019; Solís-García et al., 2021), we hypothesized that 1) symptomatic trees would exhibit an increased dominance of copiotrophic bacterial taxa in their rhizosphere due to root necrosis, which would be reflected at the compositional and functional level, and 2) PRR would induce the expression of stress-related genes in rhizosphere bacterial communities of symptomatic trees.”

Methods:

L116: Is this soil rhizospheric or bulk?

R: We now specify that the soil samples used for physicochemical analyses were taken from the rhizosphere soil (sensu lato) to obtain a soil volume large enough to perform all chemical analyses.

L143, clean version of the revised manuscript: “Three randomly selected trees per condition (asymptomatic and PRR-symptomatic) were chosen in different areas of the orchard to collect 500 g of rhizosphere soil (sensu lato, i.e., soil around the fine roots of the tree) at approximately 100 cm away from the tree trunk, for background chemical characterization.”

L188: I suggest including how the Venn diagrams were calculated. What were the abundance and prevalence thresholds to determine if an ASV is present within a group?

R: The prevalence threshold was applied before all statistical analyses. We added this information to the Methods section and specified that taxa with prevalence <5% were not considered for downstream analyses.

L209, clean manuscript: “Taxa with low prevalence (threshold < 5%) were excluded from further analysis”.

We did not use any abundance filter to build the Venn diagrams. The construction of Venn diagrams was made considering all ASVs regardless of their abundance. A list was made with the unique ASVs per condition, which was used as input data for the ggven package to obtain a graphical representation of the unique and shared ASVs. We added these details to the corresponding Methods section.

L218: “Venn diagrams were built to visualize the unique and shared bacterial ASVs in each condition with the ggven package in R (Gao et al., 2021), using all ASVs regardless of their abundance.”

R and packages should have citations.

R: The R package was cited L201. We verified that all packages included their respective reference.

SRA number does not give hits in NCBI

R: The data was released on February 19, 2026.

Results:

L260: I found the Venn diagram analysis difficult to interpret, especially without describing how it was calculated. Only 3.8% ASVs are shared, which indicates that the majority of ASV are unique to each condition. This makes it seem that these communities are very different; however, there is no significant difference in their diversity and structure. Also, this finding was not discussed further; therefore, I would reconsider its relevance in the work.

R: We added methodological details regarding the construction of the Venn diagrams. To avoid any confusion and for more consistency with the DeSeq analysis, we decided to remove former Fig 2A (Venn diagram at the genus level) and to only keep the Venn diagram at the ASV level.

The vast majority of ASVs are indeed exclusive of one tree condition (asymptomatic vs. symptomatic). We now discuss that the lack of detected differences in community structure (PERMANOVA) may be due to the large data dispersion of bacterial communities in the rhizosphere of symptomatic trees, as shown by the betadisper analysis, and to data variability intra- and inter-replicates. We compare our findings with those from other studies on root rot, which also report a lack of effect of the responsible pathogen on alpha and beta diversity metrics but highlight changes at the compositional level in rhizosphere bacterial communities. Please refer to the clean version of the revised manuscript L411.

“The lack of differences in alpha-diversity or beta-diversity metrics between rhizosphere bacterial communities associated with asymptomatic and symptomatic trees has been previously described in avocado as well as other crops, such as pecan trees (Muñoz-Castellanos et al., 2024; Solís-García et al., 2021). Consistently with our findings, these authors reported that, despite the lack of effects of the disease on bacterial diversity metrics, root rot in avocado or pecan trees induced compositional changes in bacterial communities. They argue that intra- and inter-replicate variability (Muñoz-Castellanos et al., 2024) as well as larger data dispersion in the symptomatic condition (Solís-García et al., 2021) could have obscured possible differences in terms of community structure between tree conditions, as may have occurred in the present study. Here, we show that a high proportion of the detected ASVs were exclusive of symptomatic or asymptomatic trees. Given that we did not find significant changes in soil chemical characteristics between the tree conditions, these shifts in composition were likely induced by the presence of the pathogen”.

L274: This section describes the diversity analysis of the meta-transcriptomic approach; however, there is no description in the methods about this. Also, Supplementary Figure 2 shows only 6 samples analyzed, while the methods described 19 samples processed. It is not clear whether this figure represents the transcript diversity or the taxonomic diversity inferred from the transcriptome.

R: Sorry for the confusion. Nineteen rhizosphere soil samples were processed for RNA extraction. As our sequencing budget was unfortunately limited, we only sequenced six sequencing libraries, three per tree condition. For that reason, we distributed the 19 RNA samples into six equimolar samples that were shipped for Illumina sequencing. This is now clarified:

L183: “The nine samples with intact RNA retrieved from asymptomatic trees were randomly distributed into three equimolar pools and the 10 samples retrieved from PRR-symptomatic trees were similarly distributed into three equimolar pools. Six libraries were then built from the six pools at Macrogen, Inc”.

Supplementary Figure 2 represents the taxonomic diversity inferred from the metatranscriptome. We added the description of the methods to the corresponding Methods section and reformulated the caption of Suppl. Fig. 2 to specify the number of replicates and avoid any confusion.

L253: “To determine the taxonomic diversity of active bacterial communities, Shannon and Simpson indices were calculated with the diversity function of the vegan package in the R software. Statistical differences between conditions were tested utilizing the Wilcoxon test.”.

Supplementary Figure 2. Taxonomic diversity and structure of the active bacterial community in the rhizosphere of asymptomatic and PRR-symptomatic avocado trees. A) Shannon and Simpson indices of active rhizobacterial communities associated with asymptomatic and PRR-symptomatic avocado trees, as inferred from the metatranscriptome. The W and P values were calculated with the Mann-Whitney-Wilcoxon test. B) Principal component analysis (PCA) showing the functional structure of asymptomatic and PRR-symptomatic avocado trees retrieved from expression data across all the metatranscriptome (three RNA sequencing libraries per tree condition).

L311-317: The high abundance of Mycoplasma haemophelis transcripts, which is an obligate parasite of cats, is puzzling. Does your annotation strategy really allow you to classify these genes to a bacterial species? Or is it a misinterpretation?. Would the following be a better description of these results? --The most significantly enriched transcripts were homologous to genes from Mycoplasma haemofelis?--. If not, would you consider Mycoplasma haemophelis as rhizospheric or plant-associated?

R: The annotation strategy was implemented with the NCBI RefSeq bacteria, a database which has a high quality control of the genomes that are part of the annotation (Goldfarb et al., 2025). We are thus confident that the annotation can be performed at the species level.

Reference:

Goldfarb T, Kodali VM, Pujar S, Brover V, Robbertse B, Farrell CM, et al. 2025. NCBI RefSeq: reference sequence standards through 25 years of curation and annotation. Nucleic Acids Research, 53, D243–D257. https://doi.org/10.1093/nar/gkae1038

The presence of Mycoplasma has been reported in several cropping systems such as rice or sesame. This presence has been attributed to animal and human interaction with the crop, and it is possible that cats may have been in contact with the orchard, providing a source for horizontal bacterial transmission.

References:

Roman-Reyna, V., Pinili, D., Borja, F.N., et al. (2020). Characterization of the Leaf Microbiome from Whole-Genome Sequencing Data of the 3000 Rice Genomes Project. Rice, 13, 72.

Wang, R., Lyu, F., Lyu, R., He, J., & Wei, L. (2025). Applying an appropriate rate of rice husk biochar improves the microbial community structure of rhizosphere soil in continuously cropped sesame. Scientific Reports, 15(1), 35115.

L324-330: The authors describe the functions performed by the most enriched bacterial genera in the PRR-symptomatic trees; however, I consider that these findings lack a point of comparison. Are there any genes in these categories also enriched in the asymptomatic trees? Are these genes associated with the same genera in asymptomatic trees? What are the functions of the genera enriched in asymptomatic trees? My intention is not for the authors to answer these questions but for them to better frame these findings in the context of a hypothesis.

R: The enriched transcripts in the categories graphed in Figure 5 are associated with PRR-symptomatic avocado trees, and are only enriched in this condition. A few of these categories are enriched in the asymptomatic trees but were not graphed, as we chose to focus on discussing functional categories associated with the symptomatic condition. We clarify in caption from Figure 5 that “Each panel enlists the top functions for each bacterium with Log2FoldChange values > 0 (enriched in the PRR-symptomatic condition, as compared with the asymptomatic condition) and a P-value < 0.05”. Moreover, to provide some context, we added a Supplementary Table (Suppl. Table S2) that enlists the enriched categories (FoldChange value > 0 and a significant P value) in the most active bacterial taxa for the PRR-symptomatic condition.

Discussion:

L344-346. It would be great to have more arguments or examples on how these findings could contribute to the development of microbiome-based strategies for plant protection.

R: We reformulated this section to clarify that our hypotheses were confirmed and that we indeed found 1) an increased carbohydrate metabolism in the rhizosphere of symptomatic trees and 2) transcripts related with a stress response expressed in the most active bacterial taxa in symptomatic trees (L375, clean manuscript). Since some of these active taxa are known plant growth promoters and pathogens antagonists (Rhizobium, Arthrobacter, Burkholderia…), we hypothesized that they may have been recruited by the plant upon infection and may constitute potential antagonists for the biocontrol of PRR that could be included into the integrated management of PRR in avocado.

L350-362. The authors argue that the differences in substrate preference by bacteria and pathogens could explain the absence of changes in community diversity in the diseased trees. However, these arguments might be ignoring the space these microorganisms are occupying, the bacteria in the rhizospheric soil particles, and the pathogen in the root (surface and vascular tissues?). Would resource competition apply under these circumstances?

R: In this study, we focused on rhizosphere soil bacterial communities. Although P. cinnamomi ends up colonizing external and internal root tissues, it is a soil-borne pathogen and was detected in the rhizosphere soil samples that we retrieved from the symptomatic trees. We therefore think that resource competition between the pathogen and rhizosphere soil fungal communities may apply. Our complementary study (in preparation) investigating the effect of P. cinnamomi on rhizosphere fungal communities in the same orchard confirms that the pathogen affects fungal community structure. To further strengthen this point, we have added some references in the Discussion that support the fact that root rot is usually associated with stronger impacts on rhizosphere fungal communities than on bacterial communities, due to nutrient and niche competition (L393).

Additional references:

Díaz-Cruz, G. A., & Cassone, B. J. (2022). Changes in the phyllosphere and rhizosphere microbial communities of soybean in the presence of pathogens. FEMS Microbiology Ecology, 98(3), fiac022.

Jiang, J., Yu, M., Hou, R. et al. (2019). Changes in the soil microbial community are associated with the occurrence of Panax quinquefolius L. root rot diseases. Plant Soil, 438, 143–156.

Muñoz-Castellanos, L. N., Avila-Quezada, G. D., Sáenz-De La Riva, G., Salas, E., Muñoz-Ramírez, Z. Y., & González-Escobedo, R. (2024). Revealing microbial patterns in the rhizosphere of pecan trees asymptomatic and symptomatic for Texas root rot using a high-throughput sequencing approach. Rhizosphere, 29, 100833.

L353: The bacterial diversity and structure were not significantly different between symptomatic and asymptomatic trees. In my opinion, this does not necessarily mean that there is a “lack of effect” of P. cinnamomi (L353), because the authors are not evaluating the infection process alone (e.g., a controlled infection experiment) but already diseased trees (in the context of their environment). It seems that the avocado bacterial rhizospheric communities are resilient toward the disease, rather than the pathogen not having an effect on diversity and structure.

R: Thank you for your comment. We agree that the lack of observed effect on bacterial community diversity or structure does not imply a lack of biological effect, as seen in the differential enrichment of taxa in the symptomatic tree condition and the effect of the disease on bacterial functions (DeSeq and Venn diagram analyses). We have reformulated this section accordingly (L387, clean manuscript). We have indeed carried out a controlled infection under greenhouse conditions (data presented in Reverchon et al., 2023, the full manuscript is in preparation) that shows that bacterial alpha diversity is not affected by P. cinnamomi during the disease process (infection up to tree death), nor bacterial community structure (no significant differences in the PERMANOVA).

L362-363: I don't agree that the findings suggest this. This line is based on the author's argumentation of resource competition, not based on the actual findings. I suggest rephrasing this part.

R: This was a misunderstanding, as by “findings” we meant “findings from the literature”. We have rephrased this sentence accordingly to replace “findings” by “reports” and have strengthened our point by adding the following sentence to this section:

L393: “Root rot symptoms in general have shown to produce stronger alterations in fungal than bacterial communities in the rhizosphere (Díaz-Cruz and Cassone, 2022; Jiang et al., 2019; Muñoz-Castellanos et al., 2024), which was attributed to nutrient and niche competition”.

L373: The authors argue that the enriched ASV from asymptomatic trees might be contributing to maintaining the host health based on similar taxa in other pathosystems. While I agree, I think this should be toned down, because these taxa are not absent from symptomatic trees (just less abundant) and might be contributing to plant health there too. Also, there are alternative explanations. Would you consider that these taxa are actually the most sensitive to the PRR disease because they have lower abundance in the symptomatic trees compared to asymptomatic trees? Filamentous pathogens are known to secrete antibacterial effectors with different degrees of specificity, which might explain this pattern.

R: You are absolutely right and it is possible that enriched taxa in the asymptomatic tree rhizospheres may be, in fact, more susceptible to the presence of the pathogen. We added a mention of this possible alternative mechanism. We have tuned down our statement that they contribute to plant health, although, in the case of Rhodanobacter and Variovorax, previous reports seem to support their biocontrol potential against soil-borne pathogens. We also added some references on the chitinolytic and cellulolytic enzymes produced by Chryseolinea, which support its possible antagonistic activity.

L440: “Overall, these studies suggest that the presence of these bacteria in the rhizosphere of asymptomatic avocado trees may contribute to maintaining the health of their host. Alternatively, the enriched taxa in the rhizosphere of asymptomatic avocado trees may be more susceptible to the presence of the pathogen than other rhizosphere bacterial taxa (Chavarro-Carrero et al., 2024), although previous reports of antagonistic activity displayed by Variovorax or Rhodanobacter against soil-borne pathogens support a possible contribution to plant defense”

L450: “...; further studies are thus necessary to elucidate whether Chryseolinea could assist trees in mitigating P. cinnamomi infection. Although little studied, this genus, as a member of the Cytophagales, has the capability to produce cellulolytic and chitinolytic enzymes, making it a suitable candidate to antagonize the growth of soil-borne fungal and oomycete pathogens (Glavina et al., 2010; Tan et al., 2023).

L436. The transcriptomic findings suggest that the bacterial communities are reacting to Carbon released from the roots. This is based on the enrichment of 3 proteins: from the TCA, 2-MC and the 4irosn-4sulfur domain. I wonder if this is enough evidence to support one of the main conclusions of this work. Can the authors show evidence that these genes are co-expressed with other important genes for carbon metabolism? Is there experimental evidence about the differences in carbon composition in the rhizosphere of these plants under disease?

R: Although we did not perform physicochemical analyses from the rhizosphere soil to compare carbon pools between asymptomatic and PRR-symptomatic conditions, we found at least 13 transcripts related to the carbohydrate metabolism in the rhizosphere of symptomatic trees, from a list of 33 enriched genes. The whole list showing significantly enriched genes for both conditions has been included as Supplementary Table S1.

L496: “The significant enrichment of the 4iron-4sulfur cluster (COG), another key player in carbon metabolism, and of at least 10 more transcripts related to the carbohydrate metabolism in the mRNA pools from the rhizosphere of infected trees, indicates that active rhizosphere bacteria in symptomatic avocado trees are likely taking advantage of the C resources released by root rot”.

There seems to be a small disconnect regarding stress-response results and discussion. The abstract indicates that the most active genera in PRR-symptomatic trees have transcripts of stress response and signaling (L37), while the results only indicate that they had transcripts associated with stress conditions (L326), but it is unclear which ones in Figure 5. Moreover, the discussion mostly highlights the stress genes that are enriched in the asymptomatic trees and depleted in PRR trees, indicating a loss of resilience (L454-456). The authors do not really discuss the results in L37 and L326 that seem to be part of the main findings; they mostly discuss other microbial processes like carbon metabolism and chemotaxis in page 21 and 22. Do these findings indicate that the stress response is important in both conditions?

R: As discussed (L505), stress responses in the global expression profile of the community of asymptomatic tree pools are overrepresented; we hypothesize that the loss of this global response to stress in infected trees may represent a loss of resilience from the rhizosphere bacterial community. Interestingly, when we analyzed the genes expressed by the most active genera in infected trees, we detected that some of them belonged to the category of stress response, suggesting that these most active taxa kept the ability to respond to adverse conditions (Fig. 5). To clarify this point, we added Supplementary Table S2, which enlists the genes (and their functional categories) that are overrepresented in the most active genera with a FoldChange value > 0 and a significant P value. These overrepresented genes include genes related to stress response, carbohydrate metabolism, chemotaxis and signaling.

We added a sentence to the Discussion to highlight that response to stress is indeed important in both conditions, either at the community level (asymptomatic condition) or at the active taxon level.

L562: “Although stress responses are less represented in the global expression profile of the mRNA pools from PRR-symptomatic trees than in those from asymptomatic trees, possibly indicating a loss of resilience of rhizosphere bacterial communities upon tree infection, the presence of genes belonging to the category of stress response expressed by the most active bacterial genera in infected trees suggest that these most active taxa kept the ability to respond to adverse conditions”.

Reviewer 2

You present a substantial multi-omics dataset, and the metabarcoding and metatranscriptomic workflows appear carefully executed. However, the manuscript has a weakness based around the study design and what I believe is an overreach in interpreting those results. You frame the results as evidence for plant-driven recruitment (“cry-for-help”) and microbiome-mediated mitigation, yet the work uses a single cross-sectional sampling event in one orchard, with no manipulation and no tracking of change through time. A revised manuscript needs tighter alignment between claims, design, and statistics (less over reaching).

R: We have tuned down our statements regarding the cry-for-help mechanisms underlying the enrichment of some taxa in the asymptomatic trees, as we indeed did not validate this potential explanation with downstream experiments. We also clarify that this study is based on a single sampling and that longer-term, broader-scale studies should aim at confirming our results. We also added more references regarding the reported benefits of some taxa enriched in the symptomatic condition, to support our claim that they may constitute promising biocontrol agents (albeit experimental confirmation). The details of the changes made to the manuscript are reported below, in our answers to the Reviewer´s specific comments.

1. You interpret symptomatic versus asymptomatic differences as active recruitment and mitigation. A single time point observational comparison does not test recruitment, directionality, or mitigation. It only supports association. Please either add experimental evidence (time series across disease progression, inoculation, exudate manipulation, targeted functional assays), or reframe the manuscript explicitly as a descriptive association study and remove mechanistic language from the abstract, results interpretation, and conclusion.

R: We have rewritten the manuscript to tune down our conclusions and remove mechanistic explanations from the text, emphasizing that our study constitutes a snapshot of the effects of PRR on the avocado rhizosphere bacteriome. As this comment is associated with the following comment regarding the rephrasing of recruitment of beneficial taxa by the plant or the mitigation of the disease by beneficial bacteria, we detail below, as a response to the second comment, the specific modifications made to the manuscript.

2. The manuscript repeatedly presents recruitment as a demonstrated outcome. An alternative explanation fits the design better, namely resource release and altered chemistry after root damage or necrosis, which would favour opportunistic taxa without requiring plant-driven recruitment. Please treat recruitment as a hypothesis with clear predictions, and rewrite sections that currently state recruitment or mitigation as findings.

R: We added some hypotheses to the Introduction as requested by Reviewer #1, where we predict the enrichment in copiotrophic taxa in the rhizosphere of symptomatic trees, due to the release of nutrients following root rot (L116, clean manuscript). We agree that the recruitment of beneficial microorganisms in the rhizosphere of symptomatic trees is a potential explanation based on defense priming or antagonism reports from the literature, and have rephrased the manuscript to clearly state that downstream experimental validation is required to verify this potential explanation.

Hypotheses, L114: “Based on findings from previous studies investigating the effects of root rot on rhizosphere microbiomes (Magagula et al., 2025; Shu et al., 2019; Solís-García et al., 2021), we hypothesized that 1) symptomatic trees would exhibit an increased dominance of copiotrophic bacterial taxa in their rhizosphere due to root necrosis, which would be reflected at the compositional and functional level, and 2) PRR would induce the expression of stress-related genes in rhizosphere bacterial communities of symptomatic trees”.

Discussion, L370: “By investigating for the first time the impact of P. cinnamomi on the active bacteriome in the avocado rhizosphere, we showed that the enrichment of some bacterial genera in the PRR-symptomatic avocado trees may be related to the release of resources by root rot and that the active bacteria could be taking advantage of these released nutrients, as the metatranscriptomic analysis highlighted an activated carbohydrate metabolism in the rhizosphere of PRR-symptomatic trees. These results confirmed our first hypothesis regarding the enrichment of copiotrophic taxa in the rhizosphere of infected trees”.

Discussion, L384: “As our findings are based on a single-event, single-location sampling, further replicated field studies should aim at confirming the identification and validation of such beneficial microorganisms.”

Discussion, L440: “Overall, these studies suggest that the presence of these bacteria in the rhizosphere of asymptomatic avocado trees may contribute to maintaining the health of their host. Alternatively, the enriched taxa in the rhizosphere of asymptomatic avocado trees may be more susceptible to the presence of the pathogen than other rhizosphere bacterial taxa (Chavarro-Carrero et al., 2024), although previous reports of antagonistic activity displayed by Variovorax or Rhodanobacter against soil-borne pathogens support a possible contribution to plant defense”.

Discussion, L473: “Downstream experimental validation is warranted to elucidate whether enriched taxa in the rhizosphere of infected trees result from an increase in available carbohydrates or from a plant defense response, as our study constitutes a single time-point comparison of bacterial communities associated with asymptomatic and PRR-symptomatic trees. To narrow these future experiments, we performed further functional analyses to dig deeper into the saprotrophic activity and/or potential of these bacterial taxa for disease mitigation.”

Discussion, L526: “Most of these taxa have been associated with plant growth promotion and disease suppression, which suggests they could have been recruited by the plant upon infection to assist its defense response, although we cannot discard the possibility that these taxa were attracted by the nutrient release following root necrosis.”

3. With one orchard and one sampling date, spatial structure and management variation pose major confounding risks. Symptomatic and asymptomatic trees might differ in soil, irrigation, microtopography, rootstock, cultivar, age, or prior management. At present, the manuscript does not convince the reader that PRR status explains most of the observed differences.

Please include a clear description of orchard layout and sampling strategy, including how you matched symptomatic and asymptomatic trees. I would expand the soil characterization to represent all sampled trees, not a small subset and then test to see if the soil characteristics observed are diving aby of the changes ie. Incorporate soil covariates and spatial structure into the community analyses, and report how these factors shift conclusions.

R: Symptomatic and asymptomatic trees were randomly located within the orchard, and did not correspond to a specific zone within the orchard. Although it is possible that soil parameters may vary at the micro-scale, soil within the orchard was homogeneous in terms of physico-chemical properties. Cultivar was similar (Méndez cultivar), trees were approximately 8-15 years old and organic management practices were consistent across the orchard.

A more detailed description of the orchard has been included in the manuscript.

L126: “The selected orchard covers an area of six hectares and has an organic management. Avocado trees (Méndez cultivar) were aged between eight and 15 years old. The presence of P. cinnamomi has been recorded in recent years in the orchard. On April 2023, we randomly selected 10 avocado trees that showed symptoms of PRR according to Solís-García et al. (2021) —with defoliation, dead or chlorotic leaves, coupled with small, brittle and/or necrotic roots)— and 10 asymptomatic avocado trees, spanning the entire orchard. All trees were at least 20 m apart.”

Unfortunately, we could only perform background chemical soil characterization for three soil samples per condition. The selected trees were chosen at different points of the orchard to account for spatial heterogeneity, although we agree that it would have been optimal to perform soil characterization from all samples used for metabarcoding and metatranscriptomics. For this reason, we did not establish correlations between soil data and the metabarcoding data. As already described, we did not find differences in the measured soil chemical parameters between conditions, except for ammonium.

L143: “Three randomly selected trees per condition (asymptomatic and PRR-symptomatic) were chosen in different areas of the orchard to collect 500 g of rhizosphere soil (sensu lato, i.e., soil around the fine roots of the tree) at approximately 100 cm away from the tree trunk, for background chemical characterization.”

4. Pooling samples into a small number of libraries reduces effective replication and masks between-tree variability. I understand why, it is still expensive and you may not have had enough RNA for the sequencing. Please state the effective sample size for transcriptomics clearly and carry that through the analysis and wording. Interpret gene expression patterns as pool-level signals rather than tree-level (individual) biology.

R: Thank you for your comment, which is related to a comment also raised by Reviewer #1.

As we explained, nineteen rhizosphere soil samples were processed for RNA extraction. Due to financial limitations, we only sequenced six sequencing libraries, three per tree condition. For that reason, we distributed the 19 RNA samples into six equimolar samples that were shipped for Illumina sequencing. This is now clarified:

L183: “The nine samples with intact RNA retrieved from asymptomatic trees were randomly distributed into three equimolar pools and the 10 samples retrieved from PRR-symptomatic trees were similarly distributed into three equimolar pools. Six libraries were then built from the six pools at Macrogen, Inc”.

Supplementary Figure 2. Taxonomic diversity and structure of the active bacterial community in the rhizosphere of asymptomatic and PRR-symptomatic avocado trees. A) Shannon and Simpson indices of active rhizobacterial communities associated with asymptomatic and PRR-symptomatic avocado trees, as inferred from the metatranscriptome. The W and P values were calculated with the Mann-Whitney-Wilcoxon test. B) Principal component analysis (PCA) showing the functional structure of asymptomatic and PRR-symptomatic avocado trees retrieved from expression data across all the metatranscriptome (three RNA sequencing libraries per tree condition).

We also clarify that transcript analysis is made from mRNA pools rather than from mRNA from each individual rhizosphere. See for example:

L483: “We found more enriched pathways in the mRNA pools from the rhizosphere of PRR-symptomatic trees than in those from asymptomatic avocado trees”.

L488: “The reiterative enrichment of the aconitate hydratase pathway in mRNA pools from infected trees in both COG and KEGG annotations suggests that carbohydrate metabolism is stimulated in the rhizobacterial communities associated with PRR-symptomatic trees”.

L496: “The significant enrichment of the 4iron-4sulfur cluster (COG), another key player in carbon metabolism, and of at least 10 more transcripts related to the carbohydrate metabolism in the mRNA pools from the rhizosphere of infected trees, indicates that active rhizosphere bacteria in symptomatic avocado trees are likely taking advantage of the C resources released by root rot”.

L519: “Overall, the depletion of these transcripts in the mRNA pools from the PRR-symptomatic avocado trees could indicate a loss of resilience in the event of additional stressors, such as drought or nutrient scarcity”.

5. You report non-significant outcomes for key community-level tests, yet you use strong dysbiosis language in the interpretation and conclusion. Please align the conclusions with the statistics. If the strongest signal sits in differential abundance of specific taxa or pathways rather than whole-community separation, then focus your conclusions there and quantify effect sizes and uncertainty.

R: We agree with the reviewer and rephrased and tuned down our discussion of bacterial taxa recruitment or enrichment leading to a dysbiosis, to avoid over-interpretation of our results. As mentioned above, mechanistical explanations were removed from the manuscript and we made clear that the discussion of our results warrants further validation. We now discuss our results on the context of other studies on root rot pathogen effects on rhizosphere microbiomes and highlight that the lack of significant effect of P. cinnamomi on bacterial community alpha and beta diversity metrics may not indicate that the pathogen does not alter the rhizosphere microbiome.

As also mentioned in our responses to Reviewer #1, we clarify that the lack of detected differences in community structure (PERMANOVA) may be due to the large data dispersion of bacterial communities in the rhizosphere of symptomatic trees, as shown by the betadisper analysis, and to data variability intra- and inter-replicates. We compare our findings with those from other studies on root rot, which also report a lack of effect of the responsible pathogen on alpha and beta diversity metrics but highlight changes at the compositional level in rhizosphere bacterial communities

L411: “The lack of differences in alpha-diversity or beta-diversity metrics between rhizosphere bacterial communities associated with asymptomatic and symptomatic trees has been previously described in avocado as well as other crops, such as pecan trees (Muñoz-Castellanos et al., 2024; Solís-García et al., 2021). Consistently with our findings, these authors reported that, despite the lack of effects of the disease on bacterial diversity metrics, root rot in avocado or pecan trees induced compositional changes in bacterial communities. They argue that intra- and inter-replicate variability (Muñoz-Castellanos et al., 2024) as well as larger data dispersion in the symptomatic condition (Solís-García et al., 2021) could have obscured possible differences in terms of community structure between tree conditions, as may have occurred in the present study. Here, we show that a high proportion of the detected ASVs were exclusive of symptomatic or asymptomatic trees”.

Moreover, the functional profiling of enriched genes in the active bacterial communities, showed that symptomatic trees significantly express transcripts related to stress response, carbohydrate metabolism, chemotaxis and signaling. Supporting our interpretation of PRR as a dysbiotic event over the rhizobacterial community.

6. The title and abstract currently promise mechanistic insight and yet the neither the sampling design nor the findings support this. The conclusion reads as confirmation of recruitment and mitigation.

R: We have rewritten the abstract and conclusions accordingly. Please refer to the revised version of the manuscript.

Respectfully, we believe that the current title “Phytophthora root rot induces compositional and functional changes in avocado rhizosphere bacterial communities” is not misleading as we indeed show that P. cinnamomi modifies the relative abundance of some taxa and that of transcripts in the rhizosphere of symptomatic trees. The title does not mention mechanistic explanations such as “recruitment” or “cry-for-help”.

7. Improve resolution and font sizes, and ensure legends allow the reader to interpret the figure without referring back to the main text. Figure 1 in particular is very hard to read

R: Figure resolution has been improved. We also modified some of the figure captions to ensure more clarity, for example by specifying the number of replicates. Figure 2 was modified as per the comments made by Reviewer #1.

8. There have - i believe been quite a few microbiome studies looking at PRR - you have selected a small number to reference - the references dont have to only be for avocado if they are relavent.

Farooq, Q.U.A., McComb, J., Hardy, G.E.S.J. and Burgess, T.I., 2024. Soil amendments for management of Phytophthora root rot in avocado and their impact on the soil microbiome. Journal of Plant Pathology, 106(2), pp.439-455.

Reverchon, F., García-Meléndez, M., Guevara-Avendaño, E., Mora-Chávez, O., Solís-García, I.A., Dáttilo, W., Guererro-Analco, J.A., Méndez-Bravo, A., Monribot-Villanueva, J.L., Patiño-Conde, V. and Pineda-García, F., 2023. Shifts in the rhizosphere microbiome and exudation profile of avocado (Persea americana Mill.) during infection by Phytophthora cinnamomi and in presence of a biocontrol bacterial strain. CABI Agriculture and Bioscience, 4(1), p.23.

Balbín-Suárez, A., Jacquiod, S., Rohr, A.D., Liu, B., Flachowsky, H., Winkelmann, T., Beerhues, L., Nesme, J., J. Sørensen, S., Vetterlein, D. and Smalla, K., 2021. Root exposure to apple replant disease soil triggers local defense response and rhizoplane microbiome dysbiosis. FEMS microbiology ecology, 97(4), p.fiab031.

Giachero, M.L., Declerck, S. and Marquez, N., 2022. Phytophthora root rot: Importance of the disease, current and novel methods of control. Agronomy, 12(3), p.610.

R: Thank you for your recommendations. We have now included more references on root rot, albeit not all on avocado.

Díaz-Cruz, G. A., & Cassone, B. J. (2022). Changes in the phyllosphere and rhizosphere microbial communities of soybean in the presence of pathogens. FEMS Microbiology Ecology, 98(3), fiac022.

Jiang, J., Yu, M., Hou, R. et al. (2019). Changes in the soil microbial community are associated with the occurrence of Panax quinquefolius L. root rot diseases. Plant Soil, 438, 143–156.

Magagula, P., Swart, V., Fourie, A., Vermeulen, A., Nelson, J. H., Van Rooyen, Z., & Van den Berg, N. (2025). Avocado rhizosphere community profiling: white root rot and its impact on microbial composition. Frontiers in Microbiology, 16, 1583797. https://doi.org/10.3389/fmicb.2025.1583797

Muñoz-Castellanos, L. N., Avila-Quezada, G. D., Sáenz-De La Riva, G., Salas, E., Muñoz-Ramírez, Z. Y., & González-Escobedo, R. (2024). Revealing microbial patterns in the rhizosphere of pecan trees asymptomatic and symptomatic for Texas root rot using a high-throughput sequencing approach. Rhizosphere, 29, 100833.

Reverchon et al. (2023) is a datapaper from our complementary study on a time-course infection experiment looking at elucidating the effects of P. cinnamomi on the avocado rhizosphere microbiome throughout the disease progression. We added the reference to the “perspective” section.

**Reviews of manuscript FEMSMC-2025-067.R1**

**Reviewer 1: Victor Flores Nuñez, Albrechts University**

**Recommendation: Minor Revision**

I think the author addressed all the comments and made changes that improved the interpretation and discussion of the results. They toned down mechanistic inferences and managed to highlight the actual evidence they have.

I still have some concerns regarding the “unique” and “shared” ASVs comparison. I agree with the interpretation of the community structure results (L411). However, the evidence regarding the high proportion of “exclusive” ASV in each condition (L421) is not compelling enough, because this is influenced by the sequencing depth and sampling effort.

1)You removed all the ASV with less of 5% of prevalence (<5%), meaning an ASV was removed if it was present in less than 1 sample (5% of 20 samples = 1 sample), but this does not exclude any ASV. Perhaps you meant less than or equal than 5%, which would exclude ASVs only present in 1 sample.

2)Anyhow, if you have an ASV only present in 1 of the 10 PRR samples (or 2 of 10), your analysis will consider this ASV exclusive of PRR. However, due to the dispersion of your data, it is possible that this ASV might be just present in that sample and is unrelated to the health status of the tree, but related to other unknown factors. Therefore, you are “inflating” the unique ASV with rare taxa that might or might not be related to the factor you are analyzing. This is why several works use more strict thresholds of abundance and prevalence to calculate the unique and shared taxa.

3)Do your findings hold if you remove rare taxa (low prevalence and abundance)? If not, then your “unique” ASV might be rare taxa. This does not indicate that they are not important, but it can give you a stronger basis for your interpretations.

**Associate Editor Recommendation: Minor Revision**

In addition to the comments from the reviewer, I have listed minor language edits you may wish to consider.

I prefer not to use personal pronouns such as “we” and “our”.

The use of “up-” and “down-regulated” genes in metatranscriptomic studies is inappropriate (e.g., see line 313). Instead, what is being measured is the increased and decreased relative abundance of RNA transcripts. A second example is the term “differential expression analysis” on line 346.

The data generated in this study should be in the past tense while data from published studies should be in the present tense. I have tried to catch these edits below, but please check the manuscript carefully.

Line 51. “… decades has shed…”

Line 58. Add a comma after “composition”.

Line 64. “… pathogens produce…”

Line 77. ”… pathogens modulate…”

Line 82. ”… and ultimately, may assist…”

Line 109. ”… composition, and…”

Line 129. “… selected ten avocado…”

Line 137. In US English (as used here), it should be “mL”, “µL” etc. Please fix it throughout.

Line 166. “… 16S rRNA gene to…”

Line 184. “… the ten samples…”

Line 192. “…16S rRNA gene amplicon…”

Line 327. “… (GO0008097), and…”

Line 384. “… findings were based…”

Line 415. “Consistent with…”

Line 426. “… Udaeobacter, and…”

Line 427. ”… genus is reported…”

Line 430. ”… Variovorax is also…”

Line 431. ”… Variovorax is more…”

Line 436. ”… rhizosphere is shown…”

Line 449. ”… (2023) describe a…”

Line 459. ”… and suggest that…”

Line 460. ”… cycling, and organic…”

Line 489. ” annotations suggested that…” and ”metabolism was stimulated…”

Line 494. ”… confirmed that the 2-MC cycle was occurring too…”

Line 497. ”… least ten more…”

Line 498. ”… trees, indicated that…”

Line 499. ”… trees were likely…”

Line 502. ”… and suggest that…”

Line 510. Please fix the “o”.

Line 511. Gene names should be in italics (or capitalized if you are referring to proteins) here and throughout.

Line 527. “… which suggested they…”

Line 533. Fix the spacing between words.

Line 562. ”… responses were less…”

**EIC Decision: Minor Revision**

**Author's Response to Decision Letter for (FEMSMC-2025-067.R1)**

Dear Editor,

Please find enclosed the revised version (R2) of our manuscript FEMSMC-2025-067.R1 “Phytophthora root rot induces compositional and functional changes in avocado rhizosphere bacterial communities”, that we are re-submitting for publication in the journal FEMS Microbes. We have attended all comments made by the reviewer and by your self.

As the reviewer suggested, we have reanalyzed some data regarding to shared and exclusive taxa in the reported conditions; in consequence, Figure 2 was modified. Additionally, all the suggested language edits were attended (please refer to the point ba point answers’ section. We believe these modifications greatly improved the clarity of our manuscript.

I would like to thank you for your insightful reviews and suggestions. We hope that this revised version will be now acceptable for publication in FEMS Microbes.

Sincerely,

Dr. Alfonso Méndez-Bravo

SECIHTI – Universidad Nacional Autónoma de México

Escuela Nacional de Estudios Superiores Unidad Morelia

Laboratorio Nacional de Análisis y Síntesis Ecológica

amendezbravo@enesmorelia.unam.mx

 Answers to reviewer.

I think the author addressed all the comments and made changes that improved the interpretation and discussion of the results. They toned down mechanistic inferences and managed to highlight the actual evidence they have.

I still have some concerns regarding the “unique” and “shared” ASVs comparison. I agree with the interpretation of the community structure results (L411). However, the evidence regarding the high proportion of “exclusive” ASV in each condition (L421) is not compelling enough, because this is influenced by the sequencing depth and sampling effort.

1) You removed all the ASV with less of 5% of prevalence (<5%), meaning an ASV was removed if it was present in less than 1 sample (5% of 20 samples = 1 sample), but this does not exclude any ASV. Perhaps you meant less than or equal than 5%, which would exclude ASVs only present in 1 sample.

R: Yes, we indeed meant a prevalence of less than or equal than 5%, sorry for the confusion. This has been corrected in the revised manuscript L209 (MS with track changes).

2) Anyhow, if you have an ASV only present in 1 of the 10 PRR samples (or 2 of 10), your analysis will consider this ASV exclusive of PRR. However, due to the dispersion of your data, it is possible that this ASV might be just present in that sample and is unrelated to the health status of the tree, but related to other unknown factors. Therefore, you are “inflating” the unique ASV with rare taxa that might or might not be related to the factor you are analyzing. This is why several works use more strict thresholds of abundance and prevalence to calculate the unique and shared taxa.

R: We constructed the Venn diagram using a stricter threshold, i.e., a prevalence value of 10%, as recommended.

Indeed, the number of exclusive taxa decreased compared with the Venn diagram originally presented in Figure 2A. This did not affect alpha and beta diversity statistics and, as previously, we did not detect significant differences between tree conditions. The dispersion of our data (as shown in the NMDS) was similar when applying this stricter threshold, and we still found more dispersion in the symptomatic samples than in the asymptomatic ones. Also, the differential abundance analysis carried out with DESeq2 displayed the same results as those obtained with the prevalence threshold of 5%. This is probably because DESeq2 considers a criterium of variance in abundance across samples and overall abundance levels, and implements the Benjamin-Hochberg adjustment to correct the false discovery rate.

3) Do your findings hold if you remove rare taxa (low prevalence and abundance)? If not, then your “unique” ASV might be rare taxa. This does not indicate that they are not important, but it can give you a stronger basis for your interpretations.

R: We carried out the Venn diagram analysis with a prevalence threshold of less than or equal than 5% and an additional filtering step removing taxa with low relative abundances (<0.005%, as recommended by Bokulich et al. (2013)). Although it did not affect alpha and beta diversity metrics or the DeSeq2 analysis (see our answer to comment 2 for explanations), it also decreased the number of exclusive bacterial taxa of each tree conditions. After consideration, we decided to add this Venn diagram as a new panel for Figure 2, and we added some discussion regarding rare taxa.

R: Methods, L218: “Venn diagrams were built to visualize the unique and shared bacterial ASVs in each condition with the ggven package in R (Gao et al., 2021), using all ASVs regardless of their abundance and, subsequently, considering only those taxa with a relative abundance larger than 0.005%, as recommended by Bokulich et al. (2013)”.

R: Results, L.296 (R2 manuscript with track changes): “From the 25,862 ASVs, only 872 (3.4 %) were shared between the asymptomatic and PRR-symptomatic trees (Figure 2A). When removing rare taxa (i.e., those which relative abundance was ≤0.005%), the proportion of shared species increased to 58.75% (Figure 2B)”.

Discussion, L425: “Here, a high proportion of the detected ASVs that were exclusive of symptomatic or asymptomatic trees were rare taxa (relative abundance ≤0.005). Rare microbial taxa are fundamental for plant health and key for their host´s response to biotic and abiotic stressors (Hol et al., 2010; Jousset et al., 2017; Bejarano-Bolívar et al., 2021). However, they may also be highly sensitive to environmental perturbations, which may explain the turnover of exclusive taxa in the rhizosphere of asymptomatic and PRR-symptomatic trees (Xu et al., 2021; Sun et al., 2022).”

Associate Editor

Comments to the Author:

In addition to the comments from the reviewer, I have listed minor language edits you may wish to consider.

I prefer not to use personal pronouns such as “we” and “our”.

R: We have revised the manuscript accordingly. We only kept the active voice to mention our objectives and hypotheses at the end of the Introduction.

The use of “up-” and “down-regulated” genes in metatranscriptomic studies is inappropriate (e.g., see line 313). Instead, what is being measured is the increased and decreased relative abundance of RNA transcripts. A second example is the term “differential expression analysis” on line 346.

We reformulated as suggested by the Editor throughout the text.

The data generated in this study should be in the past tense while data from published studies should be in the present tense. I have tried to catch these edits below, but please check the manuscript carefully.

We have revised the manuscript accordingly.

All other edits have been addressed, and the text was modified as suggested.

**Reviews of manuscript FEMSMC-2025-067.R2**

**Reviewer 1: Victor Flores Nuñez, Albrechts University**

**Recommendation: Minor Revision**

I have no additional comments.

**Associate Editor Recommendation: Accept**

**EIC Decision: Accept**
